# Evaluating Underground CO_2_ Storage Potential across Sedimentary Facies of the Michigan Basin

**DOI:** 10.1021/acsomega.6c08038

**Published:** 2026-10-05

**Authors:** Nimarta Kaur, Christina Miller, Pablo Sanchez, Emyline Kirda, Alberto Gutiérrez-Vegad, Santiago Aparicio, Ashley Scott, Autumn Haagsma, Ahmad Al-Bodour, Mert Atilhan

**Affiliations:** † Department of Chemical and Paper Engineering, 4175 Western Michigan University, Floyd Hall, 4601 Campus Drive, Kalamazoo, Michigan 49008, United States; ‡ Department of Mechanical Engineering, San Diego State University, 5500 Campanile Drive, San Diego, California 92182, United States; § Department of Civil Engineering, 14589 University of Illinois Urbana−Champaign, 205 North Mathews Avenue, Urbana, Illinois 61801, United States; ∥ Departamento de Química, 16725 Universidad de Burgos, Plaza Misael Bañuelos s/n, 09001 Burgos, Spain; ⊥ Michigan Geological Repository for Research and Education (MGRRE), Western Michigan University, 5272 West Michigan Avenue, Kalamazoo, Michigan 49008-5731, United States; # Michigan Geological Survey, Western Michigan University, 5272 West Michigan Avenue, Kalamazoo, Michigan 49008-5731, United States

## Abstract

Rising atmospheric CO_2_ concentrations demand scalable and geologically reliable carbon capture and storage solutions. The Michigan Basin, with its well-characterized Paleozoic sedimentary sequences and proximity to major industrial emission sources, represents a promising target for underground CO_2_ storage. This study systematically evaluates the CO_2_ storage potential of nine distinct sedimentary rock facies retrieved from the Consumers Brine Disposal 139 (BD-139) well, providing a facies-resolved framework for assessing subsurface sequestration capacity. High-pressure isochoric CO_2_ adsorption experiments were conducted across three temperature isotherms (25 °C, 35 °C, and 45 °C) and six pressure points (10–35 bar), complemented by comprehensive material characterization, including Brunauer–Emmett–Teller (BET) surface area analysis, Fourier transform infrared spectroscopy (FTIR), scanning electron microscopy equipped with energy-dispersive spectroscopy (SEM-EDS), and thermogravimetric analysis (TGA). Based on their inferred mineralogical composition and thermal behavior, the nine facies were classified into three distinct groups: silicate-rich organic-bearing rocks (Group 1), siliciclastic assemblages with a subordinate carbonate component (Group 2), and carbonate-bearing dolomitic facies (Group 3). Facies 3 exhibited the highest apparent CO_2_ uptake, 855.7 mg/g at 35 bar and 25 °C, and all facies gave heats of sorption below 60 kJ/mol, consistent with predominantly physical interactions and with favorable reversibility for storage cycling. Because the measurements were performed on pulverized material, and because the apparent uptake of the highest-performing facies exceeds the volumetric capacity of its own pore system, these quantities are reported and interpreted throughout as apparent isochoric uptake determined under identical conditions. They provide a quantitative relative ranking of the facies rather than absolute in situ storage capacities, and the bounds governing their interpretation are set out in Section 3.5.3. Complementary CO_2_ absorption experiments in synthetic brine solutions yielded a solid precipitate on depressurization, indicating a potential mineral trapping pathway under high-pressure conditions. The findings establish a direct link between facies-level geological heterogeneity and CO_2_ storage performance, offering a practical screening framework for site selection and reservoir evaluation within the Michigan Basin and analogous sedimentary systems.

## Graphical abstract

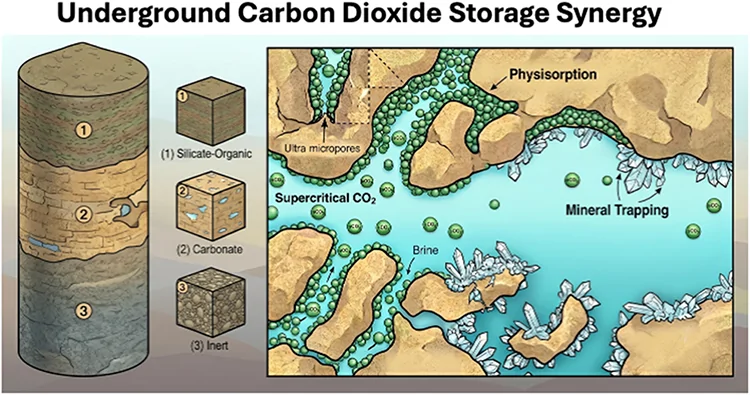

## 1 Introduction

The increasing concentration of atmospheric carbon dioxide (CO_2_) and its associated impacts on global climate systems have intensified the need for efficient and scalable carbon capture and storage technologies. The International Energy Agency reported a 0.8% increase in CO_2_ emissions in 2024, with 37.8 Gt CO_2_ emitted, thus leading to a record-high atmospheric concentration of 422.5 ppm.^1^ Due to rising global demand for energy, economic considerations, and policy dynamics, it is projected that carbon-intensive fossil fuels will likely continue to be utilized globally for several decades.^2^ Depending on the timeline for transitioning to renewable energy, it is estimated that 1000–1500 Gt of CO_2_ will need to be captured and stored to maintain an atmospheric CO_2_ concentration of 450 ppm or below.^3^

Carbon dioxide sequestration encompasses several approaches, with geological storage representing the most significant due to its large capacity and long-term stability, involving the injection of CO_2_ into subsurface reservoirs, such as depleted hydrocarbon fields and deep saline aquifers. Mineral sequestration provides an additional pathway, wherein CO_2_ reacts with divalent cations, such as Ca^2+^ and Mg^2+^, to form stable carbonate minerals, which enhances permanent storage. In contrast, terrestrial sequestration relies on biological uptake and storage in vegetation and soils, offering a more limited and variable contribution.^3^ Among the various approaches under investigation, geological sequestration has emerged as a promising long-term solution due to its capacity for large-scale CO_2_ storage within subsurface formations.^4,5^ In this context, understanding the physicochemical interactions between CO_2_ and reservoir rocks, particularly under high-pressure and variable-temperature conditions, is essential for evaluating storage capacity, retention mechanisms, and long-term stability.

Lithologic heterogeneity plays a critical role in governing the CO_2_ storage performance. Variations in mineral composition, pore structure, surface chemistry, and thermal stability can significantly influence adsorption behavior, storage capabilities, and fluid–rock interactions.^6^ Geologically, bodies of rock distinguished by a specific set of physical, chemical, and biological attributes, which collectively provide insight into the environmental conditions prevailing at the time of their deposition, are categorized as different facies.^7^ Facies-based analysis provides a systematic framework for capturing such heterogeneity, as geological facies represent distinct depositional environments and corresponding petrophysical properties. Despite its importance, the relationship between facies characteristics and CO_2_ adsorption performance remains insufficiently explored, particularly under high-pressure conditions that are relevant to subsurface sequestration.

Recent studies have highlighted the importance of surface area, mineralogy, and pore architecture in determining the CO_2_ adsorption capacity. However, most existing works have focused on engineered adsorbents or homogeneous materials. Natural rock systems, characterized by complex mineral assemblages and multiscale heterogeneity, require further investigation, although some studies have examined gas sorption in a geological setting.^8,9^ Additionally, the role of saline environments, which are ubiquitous in deep geological formations, introduces further complexity through dissolution, precipitation, and mineral trapping mechanisms that can enhance or inhibit the CO_2_ storage efficiency.^10^

In this study, nine distinct facies obtained from the BD-139 well in Southeast Michigan are systematically investigated to elucidate the relationship between geological variability and high-pressure CO_2_ storage performance. These facies, representing different depths and depositional characteristics, are analyzed in powdered form to isolate intrinsic material properties and maximize surface accessibility. The Midwestern United States is a well-suited region for geological carbon storage due to its extensive deep saline formations within Paleozoic sedimentary basins, which provide favorable reservoir conditions and effective caprock seals, combined with proximity to major industrial CO_2_ sources and the presence of naturally fractured systems that influence storage capacity and fluid migration.^11^ Michigan specifically is a strong candidate for geological carbon storage due to its well-characterized subsurface geology and proven injection performance. Effective CO_2_ sequestration requires a thorough understanding of geologic controls, including reservoir heterogeneity, structural architecture, and fluid–rock interactions, all of which are well documented within the Michigan Basin. These carbonate reservoirs, which exist at various stages of enhanced oil recovery, exhibit complex internal geometries and diagenetic modifications that influence storage capacity, pressure behavior, and overall reservoir performance. As a result, they provide an ideal natural laboratory for evaluating how geological variability affects CO_2_ storage efficiency. Furthermore, large-scale field projects conducted under the Midwestern Regional Carbon Sequestration Partnership have demonstrated successful injection and monitoring of substantial CO_2_ volumes, underscoring the technical feasibility and reliability of these formations for long-term storage.^12^

A comprehensive experimental approach is employed, combining high-pressure isochoric CO_2_ adsorption measurements with detailed material characterization techniques, including Brunauer–Emmett–Teller (BET) surface area analysis, Fourier transform infrared spectroscopy (FTIR), scanning electron microscopy (SEM) coupled with energy-dispersive spectroscopy (EDS), and thermogravimetric analysis (TGA). The adsorption experiments are conducted over temperatures ranging from 25–45 °C in increments of 10 °C and pressures ranging from 10 to 35 bar in increments of 5 bar, enabling evaluation of thermodynamic and kinetic behavior under conditions relevant to subsurface reservoirs. Complementary analyses of CO_2_ absorption in synthetic saline solutions are also performed to simulate brine–CO_2_ interactions and assess potential mineralization pathways. The inclusion of extensive morphological, compositional, and adsorption data sets allows for a multiscale interpretation of CO_2_ capture mechanisms.

By integrating adsorption performance with thermophysical and structural characterization, this work aims to classify facies according to their CO_2_ storage potential, identify key material properties governing adsorption and retention, and provide insights into the interplay between rock heterogeneity and the carbon sequestration efficiency. The findings contribute to a more nuanced understanding of natural reservoir behavior and support the development of more reliable screening criteria for geological CO_2_-storage sites.

## 2 Materials and Methods

### 2.1 Materials

Rock samples were sourced from the Michigan Geological Repository for Research and Education (MGRRE). These samples are designated as Facies #-BD-139, where BD-139 is the well name (Consumers Brine Disposal 139, located in St. Clair County, Michigan; API 21147611598000) and the # denotes the individual facies, each cut from slabbed core at different depths within the well. The BD-139 well was chosen as part of a seven-well study under DOE-funded project DE-FE0032368. Each well underwent extensive characterization, including qa/qc, elemental analysis, thin-section petrography, bulk rock mineralogy, scanning electron microscopy, core descriptions, and, in some cases, geomechanical characterization to assess reservoir and confining unit variability. BD-139 was selected due to its geographic location (region currently being assessed for CO_2_ storage), data and core coverage, and observed variability in formations of interest. Nine different facies were identified to be representative of the core, as described in Table 1 below.

**Table 1:** Summary of Facies Characteristics

facies	visual porosity	average permeability (mD)	general description	dominant lithology	formation association	total thickness (ft)	total % of core
1	0–2.5%	12.10	Pink bioturbated sandstone with green dolomitic mudstone	Sandstone and dolomudstone	Eau Claire	5.5	8%
2	None	0.41	Stylolitic lenticular pink sandstone	Sandstone	Eau Claire	6.9	11%
3	10–20%	74.95	Pink sandstone with green vertical burrows	Sandstone	Eau Claire	12.4	19%
4	2–5%	0.41	Pink and gray planar laminated sandstone	Sandstone and Siltstone	Eau Claire	1.3	2%
5	10–20%	8.29	Pink and purple dolomitic sandstone	Sandstone	Eau Claire	9.1	14%
6	5–10%	0.47	Massive green sandstone	Sandstone	Eau Claire	2.7	4%
7	None	0.32	Massive green dolomitic mudstone	Dolomudstone	Trempealeau	5.8	9%
8	0–2.5%	0.39	Laminated tan sandstone and green dolomitic mudstone	Sandstone and dolomudstone	Eau Claire and Trempealeau	14.0	21%
9	0–2.5%	0.40	Black glauconitic sandstone and green dolomitic mudstone	Sandstone and dolomudstone	Trempealeau	8.0	12%

The nine facies were sampled from the slabbed core over the depth interval of 3200–3700 ft, and the individual sampling depth of each facies is listed in Table 1. The measured bottom-hole temperature of BD-139 is estimated to be around 30–40 °C, and the corresponding formation pressure is approximately 110 bar at midinterval (105–125 bar across the sampled section), assuming normal hydrostatic conditions with a brine gradient of 0.465–0.50 psi ft^–1^. These values define the in situ conditions against which the experimental temperature and pressure ranges used in this study are assessed in Section 2.3.

CO_2_ used for experiments was of a research grade with an ultrahigh purity of 99.999% and was acquired from the company Airgas. Sodium nitrate (NaNO_3_, CAS: 7631–99–4), sodium chloride (NaCl, CAS:7647–14–5), and calcium chloride (CaCl_2_·2H_2_O, CAS: 10035–04–8) were sourced from Fisher Scientific. Iron­(III) nitrate nonahydrate (Fe­(NO_3_)_3_·9H_2_O, 99% purity, CAS:7782–61–8) and magnesium chloride hexahydrate (MgCl_2_·6H_2_O,99% purity, CAS: 7791–18–6) were sourced from Thermo Scientific.

### 2.2 Sample Preparation

To achieve a powdered texture, rock samples were placed on a clean cotton sheet and hammered by the pointed side of a rock hammer to break them gently into smaller pieces. Once the material is in smaller pieces, the samples are transferred to a ceramic grinding bowl and pounded with a ceramic pounder to a smooth, dust-like powder. Five water-soluble salts were used in order to prepare the brine solutions to reach the desired concentrations representative of the formation brines encountered in the subsurface: sodium chloride NaCl, calcium chloride CaCl_2_, magnesium chloride MgCl_2_, iron­(III) nitrate Fe­(NO_3_)_3_, and sodium nitrate NaNO_3_. All five salts were mixed in accordance with the molar ratio in 250 mL of deionized water (DI water) to achieve an aqueous brine solution.

The synthetic brine described above is a simplified ionic analogue of the formation waters of the Michigan Basin rather than a reproduction of a specific brine analysis. Iron in deep formation brines is present predominantly as Fe­(II), whereas Fe­(III) is sparingly soluble at circumneutral pH and hydrolyzes readily to ferric oxyhydroxide, and nitrate is not a significant constituent of the Na–Ca–Cl brines of the Michigan Basin. Both features are taken into account when the precipitate recovered after CO_2_ absorption is interpreted in Section 3.5.2. All specimens were prepared by the identical grinding protocol described above. The particle-size distribution was not determined instrumentally during sample preparation, and no mean particle size is assigned here; because every facies was subjected to the same procedure, differences in the measured uptake between facies are not attributable to differences in particle size.

### 2.3 High-Pressure CO_2_ Adsorption Experimental Setup

These facies-based powder samples were investigated through an in-house-built equipment for their capacity of CO_2_ adsorption (Figure 1). This custom-designed autoclave apparatus has been utilized in previous CO_2_ capture experiments.^13,14^ The adsorption experiment is carried out in a 100 mL chamber that is manufactured by Parr Instruments (model No. 4790-HP-100 mL-T-SS-VGR-5000-BTS) and is made of T316 stainless steel. An approximate mass in the range of 0.25 g of rock powder was placed at the bottom of the cell and weighed down by one cm in diameter aperture mesh. The cell has two points of opening: the introductory side, on which a gas is introduced to the chamber, and the elimination side, which exits the gas once the experiment is completed. This cell is insulated with a tube that is filled with a heat transfer fluid and is supplied from an external water bath operated by PolyScience (model No. SD07R-20-A11B). This allows the cell to reach a stable appointed temperature, at which the adsorption assessment needs to take place. The complete test procedure comprises three stages: (1) precondition, (2) experiment, and (3) purge. First, the preconditioning stage involves the CO_2_ being fed to a space, which is termed the compression chamber. The space inside this compression chamber is manually connected with a hand pump, allowing the possibility of being adjusted manually in accordance with the objective pressure for the experiment stage. It is well insulated and is regulated with a tube filled with a heat transfer fluid operated by Ai circulators (model No. C20–7 SST). Here, the CO_2_ from the gas cylinder is transported through a stainless steel tube to the compression chamber that is being recorded numerically by a pressure gauge (model no. GE-DPI Druck 104). The temperature was monitored and recorded. Once it is stable and ready, the gas is charged to the autoclave cell, proceeding to the second stage of operation, which is the experiment. The instant the CO_2_ gas is introduced to the experimenting cell, and the pressure transducer (model No. Ashcroft T2) records its actions and continues to collect the numeric data until the cell is in equilibrium. Parallelly, a transducer from the Druck Unik 5000 series (model No. PTX5072-TA-A3-CA-H0-PE) operating on the basis of analog circuitry is attached to the cell to measure the pressure drop. This Druck transducer delivers high stability, low power, and frequency responses, which allows us to measure the pressure drop with high accuracy. It is equipped with a direct current supply (model No. Instek GDP-3303S) to provide the voltage supply to generate the current while the CO_2_ gas gets in contact with this transducer. The current signals, which are analog, send the numeric data to a data acquisition hardware (DAQ model nN. NI 9207), which was used to measure the physical phenomena from the Druck transducer. The current analog signals from the transducer are captured by the DAQ and sent to a software program (LabVIEW).

**Figure 1:**
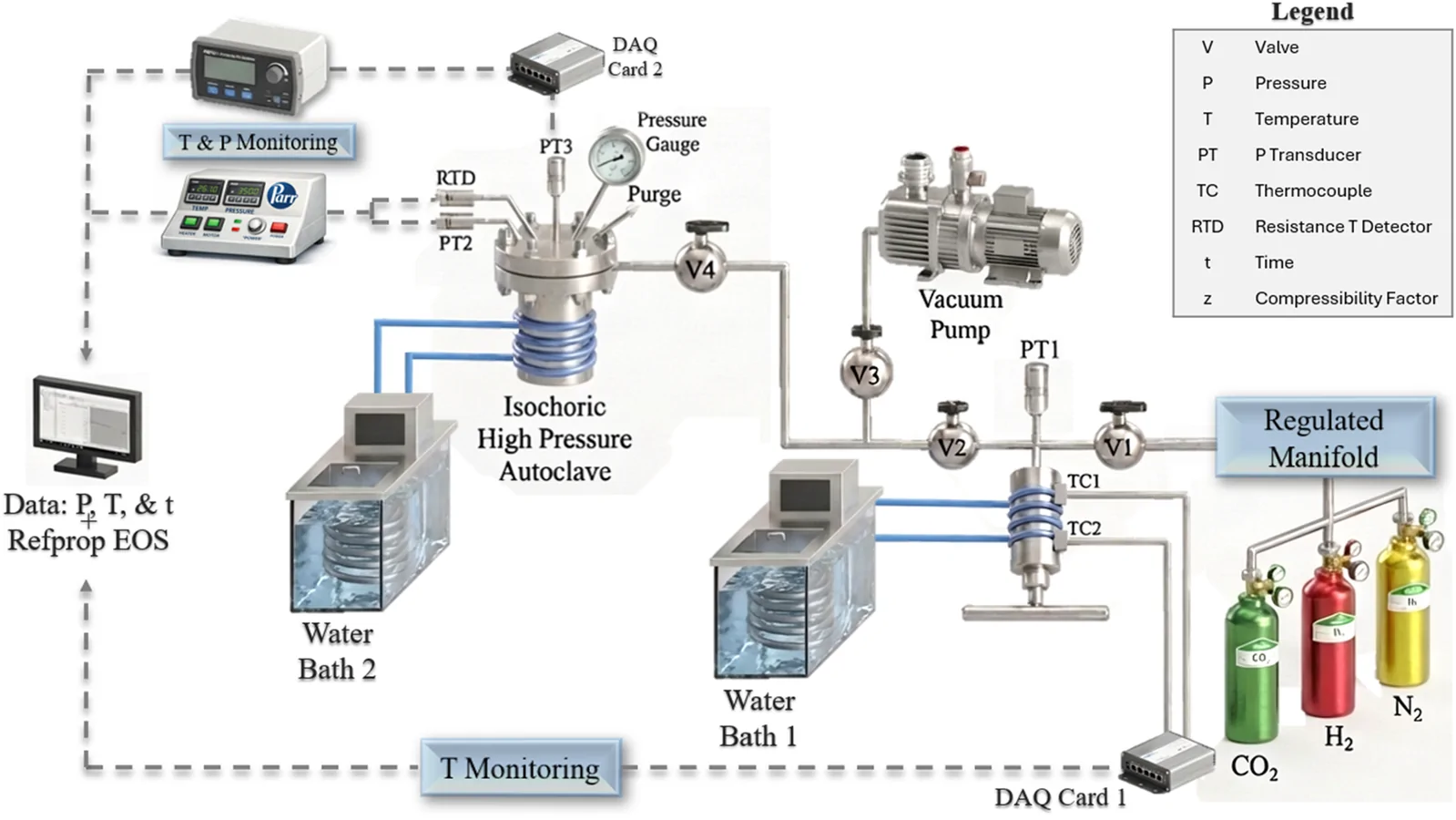
Schematic diagram of the custom-built high-pressure isochoric CO_2_ adsorption apparatus, comprising the compression chamber, Parr autoclave cell, PolyScience water bath, Druck pressure transducers, and LabVIEW-based data acquisition system. The schematic was prepared by the authors and depicts the custom-built apparatus previously described in ref 14.

From LabVIEW, a program is generated to convert these current analog signals to digital, which then displays in the pressure reading. During the gas-charging step, the valve directly connected to the high-pressure autoclave remains open, while valve V2, located approximately 1 m upstream of the autoclave, is opened to initiate gas charging. The CO_2_ therefore passes through approximately 1 m of 1/8-in. stainless steel tubing before entering the 100 mL autoclave. The charging process requires approximately 2 s to reach the maximum pressure recorded inside the autoclave. This peak-pressure point is defined as (*t* = 0), and the corresponding pressure and temperature measured by the pressure transducer and RTD, respectively, are used as the initial reference state for calculating the number of CO_2_ moles. Pressure and temperature are continuously recorded for 1 h after the peak-pressure point, allowing the system to reach thermal equilibrium and the adsorption process to approach equilibrium. The final pressure and temperature measured after 1 h are used as the final equilibrium state, and the amount of CO_2_ adsorbed is determined from the difference in the number of moles between the initial and final states. No backward extrapolation of the pressure data to (*t* = 0) is applied. The amount of adsorption that takes place is directly proportional to the pressure drop in the experimental cell. The amount of CO_2_ taken up can be calculated with a known quantity of the rocks sample, temperature, and the pressure drop. This leads to the final purge stage that eliminates the gas from the cell through an exit valve. Once the cell is empty, it is vacuumed by an external pump (Model No. Welch CRVpro8) to remove any moisture and residual gas from the cell. This vacuum will also reactivate the powdered rocks for the next adsorption experiment. The experimental CO_2_ adsorption data were collected at three temperature isotherms: 25 °C, 35 °C, and 45 °C. This temperature range brackets the expected in situ temperature at the target depth and location (approximately 30–40 °C) and therefore provides a representative range for evaluating CO_2_ adsorption under relevant subsurface conditions. All isotherms were tested at six pressure points from 10 to 35 bar in increments of 5 bar. These pressures lie below the in situ reservoir pressure at the sampled interval, approximately 110 bar at midinterval and 105–125 bar across the section, assuming normal hydrostatic conditions at a brine gradient of 0.465–0.50 psi ft^–1^. Measurements spanning 100–125 bar at 30–37 °C are therefore identified as the priority for subsequent work. The absorption experiment to determine the solubility of CO_2_ in the saline solution was conducted at two isotherms, 25 and 35 °C, with the same pressure points as the rock samples.

The temperature within the autoclave is measured with an RTD positioned adjacent to the sample, whose response time in gas at the operating pressure is determined by a step-change calibration. Because the reference state of the mass balance is defined at the pressure maximum reached approximately 2 s after the start of charging, the ability of the thermometer to resolve the temperature at that instant governs the accuracy of the initial mole count; this contribution is quantified explicitly in Section 2.4, and its consequences for the reported quantities are evaluated in Section 3.5.3.

### 2.4 Experimental Quantification for CO_2_ Adsorption

The CO_2_ uptake was quantified following the procedure described previously.^14^ The formula that incorporates the equation of state of a hypothetical ideal gas law incorporates the pressure (*P*), temperature (*T*), volume (*V*), number of CO_2_ moles (*n*), and ideal gas constant (*R*). The equation is

PV=nRT

Nevertheless, real experimental gas behavior, which is done in the lab, incorporates a deviation from the ideal gas law. The compressibility factor denoted by *z* is then integrated with the above equation. Hence, the equation is then modified to

PV=znRT

The value of *z* is estimated by REFPROP software under the National Institute of Standards and Technology (NIST) for the CO_2_ gas.^15^ The temperature (*T*) and the gas pressure (*P*) are the quantities at the initial and final states of the adsorption process. The term *V* is the constant-cell volume. The ideal gas constant is given by *R*. The initial pressure gives the initial number of moles, and the final pressure gives the final number of moles. Hence, the disparities between the initial moles and the final moles give the absolute amount of CO_2_ taken up, in moles. With the known molar mass of CO_2_, the final adsorbed value in milligrams per gram of the facies sample is calculated.

The uncertainty in the reported uptake was obtained by propagating the uncertainties in pressure, temperature, effective free volume, gas purity, and sample mass through the isochoric mass balance. A feature of the isochoric method that governs the result is that the sorbed quantity is obtained as the difference between two comparatively large mole numbers, so that the relative uncertainty of the difference is amplified by the ratio *n*/Δ*n*. For the present configuration (100 mL cell, 35 bar, 298 K, *Z* = 0.82), the cell contains *n* = 0.172 mol, while an uptake of 855.7 mg g^–1^ on a 0.25 g specimen corresponds to Δ*n* = 4.86 mmol, so the amplification factor is approximately 35. Under these conditions, an uncertainty of 0.1% in each pressure determination contributes approximately 5% to Δ*n*, an uncertainty of ±1 K in the reference temperature contributes approximately 12%, and the uncertainty in the effective free volume propagates in direct proportion to Δ*n*. Temperature is therefore the dominant term rather than pressure. Taking a pressure determination accurate to 0.1% of reading, a temperature determination accurate to ±0.5 K, an effective free volume known to 0.5%, and a sample mass known to ±1 mg, the combined standard uncertainty in the reported uptake is approximately 10% at 35 bar and approximately 18% at 10 bar, where Δ*n* is smaller and the amplification factor rises to about 65. A more consequential quantity than the relative uncertainty is the absolute resolution limit of the configuration, defined as the uptake equivalent to the combined uncertainty of the two pressure–temperature states. For a 0.25 g charge in the 100 mL cell at 35 bar, this limit is approximately 85 mg g^–1^. It is of the same order as the volumetric capacity of the specimens themselves, which is bounded at 67–97 mg g^–1^ for the most porous facies, as shown in Section 3.5.3, and it therefore sets the interpretation of the sorption data reported in Section 3.5.

### 2.5 Material Characterizations

The facies-based powder samples were characterized as described below. Characterizations such as Brunauer–Emmett–Teller (BET) surface area measurement, Fourier transform infrared spectroscopy (FTIR), scanning electron microscopy (SEM), and thermal gravimetric analysis (TGA) were used to characterize their behavior. N_2_ at 77 K, which is liquid nitrogen, is used in the BET to measure the specific surface area and the porosity with a gas adsorption technique. This was performed by Micromeritics 3Flex surface characterization physisorption analyzer, which is provided with a VacPrep 061 sample degas system unit connected to a rotary-vane Welch CRVpro2 vacuum pump for samples pretreatment. FTIR was performed on a Spectrum 3 apparatus by PerkinElmer and provides a molecular fingerprint based on an absorbed infrared light. High magnification and resolution images of the samples were obtained through Scanning Electron Microscopy and their elemental composition through energy-dispersive spectroscopy (EDS). These analyses were performed on a JEOL IT-200 InTouch microscope at an accelerating voltage of 10–20 kV to collect morphological and surficial information, with elemental composition determined by energy-dispersive spectroscopy. All SEM and EDS data reported in this work were acquired on as-prepared material prior to the sorption experiments. Thermogravimetric analysis (TGA) of the materials was performed on PerkinElmer-TGA 8000. Analyses were performed in an inert atmosphere of N_2_ to avoid any reaction occurring at high temperature. The range study was examined between 30 °C and 1000 °C at a rate of 15 °C per minute.

## 3 Results and Discussion

### 3.1 Thermogravimetric Analysis

Thermogravimetric analysis (TGA), presented in Figure 2, describes the degradation behavior of all of the facies. With this analysis, a breakdown was conducted to distinguish the facies on the basis of their thermal stability.

**Figure 2:**
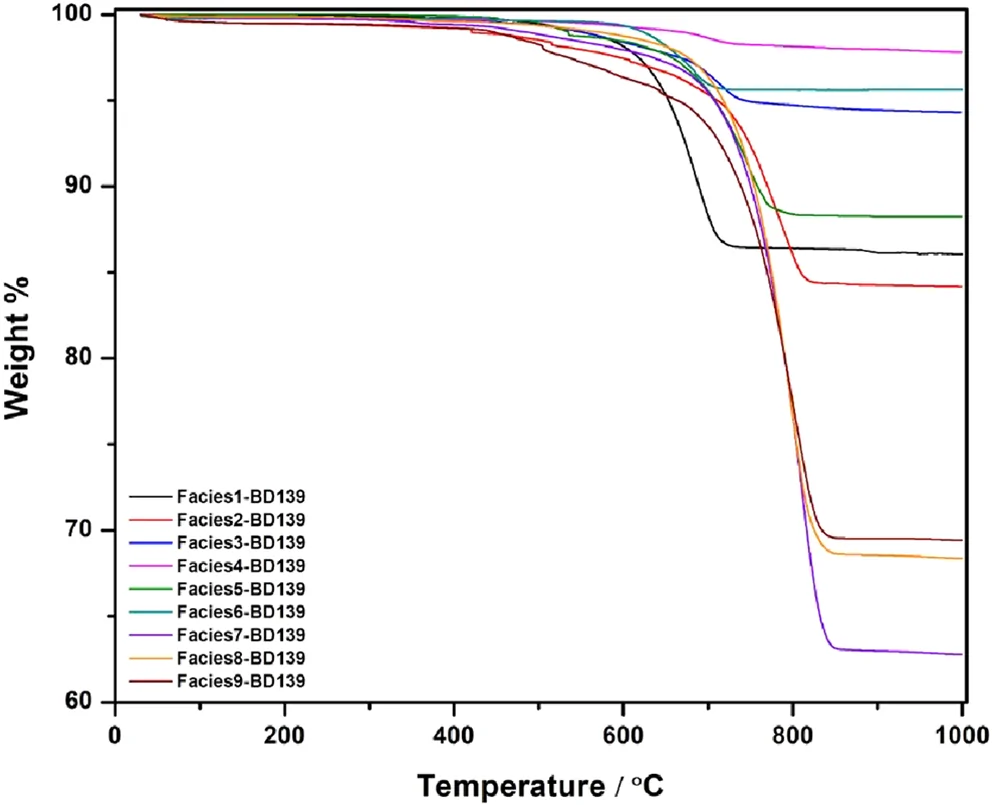
TGA profiles of nine BD-139 rock facies recorded under an inert N_2_ atmosphere from 30 to 1000 °C at a heating rate of 15 °C min^–1^. Facies are grouped according to their thermal degradation behavior: Group 1 (Facies 3, 4, and 6) exhibiting the highest thermal stability with mass loss below 2.5% up to 1000 °C; Group 2 (Facies 1, 2, and 5) displaying intermediate stability with pronounced degradation onset in the range of 580–740 °C; and Group 3 (Facies 7, 8, and 9) showing the lowest thermal resistance with mass losses approaching 37% near 800 °C. The moisture evaporation event common to all facies is observable near 100 °C, corresponding to approximately 3% mass loss.

Facies 3, 4, and 6 portray the most resistance to heat, and this falls under the category of Group 1. Group 2 consists of Facies 1, 2, and 5, and they show more degradation around 800 °C. Lastly, Group 3, which consists of Facies 7, 8, and 9, and these facies display the lowest resistance to heat around 800 °C. The moisture level for the facies can be detected around 100 °C, where the water content would vaporize. The percentage loss here for all of the facies sample is in the approximation of 3% of the sample mass.

The magnitude of the mass loss exhibited by Group 3 provides a quantitative constraint on its mineralogy. Quartz and feldspar are essentially nonvolatile under an inert atmosphere up to 1000 °C, so a loss approaching 37% is attributable to carbonate decarbonation. Complete decomposition releases 47.7% of the initial mass for dolomite [CaMg­(CO_3_)_2_] and 44.0% for calcite [CaCO_3_], so the observed loss corresponds to a carbonate content of approximately 75–85 wt %. The onset range of 680–740 °C corresponds to the first decomposition step of dolomite under an inert atmosphere, and both observations are consistent with the lithological descriptions in Table 1, which identify Facies 7, 8, and 9 as the dolomudstone-bearing facies of the sampled interval. Group 3 is therefore designated the carbonate-bearing dolomitic group. By the same argument, the negligible loss recorded for Group 1 (below 2.5% to 1000 °C) indicates a carbonate-free silicate framework, and the intermediate behavior of Group 2 indicates a siliciclastic matrix carrying a subordinate carbonate component, again in agreement with Table 1. The earlier onset observed for Facies 1 (approximately 580–620 °C) lies below the decomposition range of both calcite and dolomite and is attributed to dehydroxylation of clay minerals and glauconite, which proceeds between approximately 500 and 600 °C.

The onset temperature portrays a significant mass loss of a material, and all of the facies here exhibit a strong resistance to heat up to the range of 450 °C. Facies 4 and Facies 7 are contrasted for this thermal study, beyond 500 °C being on the opposite end of the other. Facies 4 showed the highest thermal stability with the percentage loss of about 2.5%, and Facies 7 showed the lowest stability with the percentage loss of an approximation of 37%. Facies 1 shows the earliest onset temperature in the range ∼580–620 °C. Facies 2, 7, 8, and 9 show a little longer onset at the range ∼680–740 °C. The highest stability from group 1 shows the longest onset at the temperature above ∼780 °C.

### 3.2 Fourier Transform Infrared (FTIR) Spectrometry

FTIR analysis was conducted for all species, and the spectra display analogous features within each group. Figure 3 shows Facies 3, Facies 4, and Facies 6 under the Group 1 categorization, and their spectra reveal aromatic C–H stretching (∼3050 cm^–1^), C–H stretching (∼2800–3000 cm^–1^), and strong Si–O/Al-O vibrations (∼1000–1100 cm^–1^), indicating a silicate-rich mineral matrix. The FTIR spectra strongly support the EDS findings, with intense transmittance minima around 1000–1100 cm^–1^, assigned to asymmetric Si–O–Si stretching vibrations. The high thermal stability of Group 1 facies (minimal weight loss up to 1000 °C) aligns with its silicate-rich, low-volatile composition. The peak at 2800–3000 cm^–1^ is an indication of organic matter, and it only exists in this particular group that separates their characteristics to the other groups. Group 1 (facies 3,4,6) shows the possibility of organic-rich rock samples, as indicated by the organic-matter signatures in their spectra. Group 2 (facies 1,2,5) and Group 3 (facies 7,8,9) lack organic traits in their peaks. Group 2 shows the band at ∼1450 cm^–1^, and this can be an identification of calcite/carbonate vibrations. They demonstrate that when these peaks are present, they often “mask” smaller organic signals, where Facies 1 and 2 differ from the organic traits of Facies of group 1. The Group 3 spectra are dominated by silicate framework absorption. The carbonate component indicated by thermogravimetry is not resolved as a distinct band in these spectra, the broad ν3 envelope near 1450 cm^–1^ being neither diagnostic of carbonate speciation nor readily separated from the silicate absorption; discrimination of calcite from dolomite requires the ν2 bands at 872 and 880 cm^–1^, respectively, and the ν4 bands at 712 and 728 cm^–1^, and the carbonate assignment adopted here is therefore based on the thermogravimetric response of Section 3.1 together with the core lithologies of Table 1. The remaining absorption is consistent with quartz- and feldspar-dominated assemblages containing variable clays, glauconite, and pyrite.^17–19^

**Figure 3:**
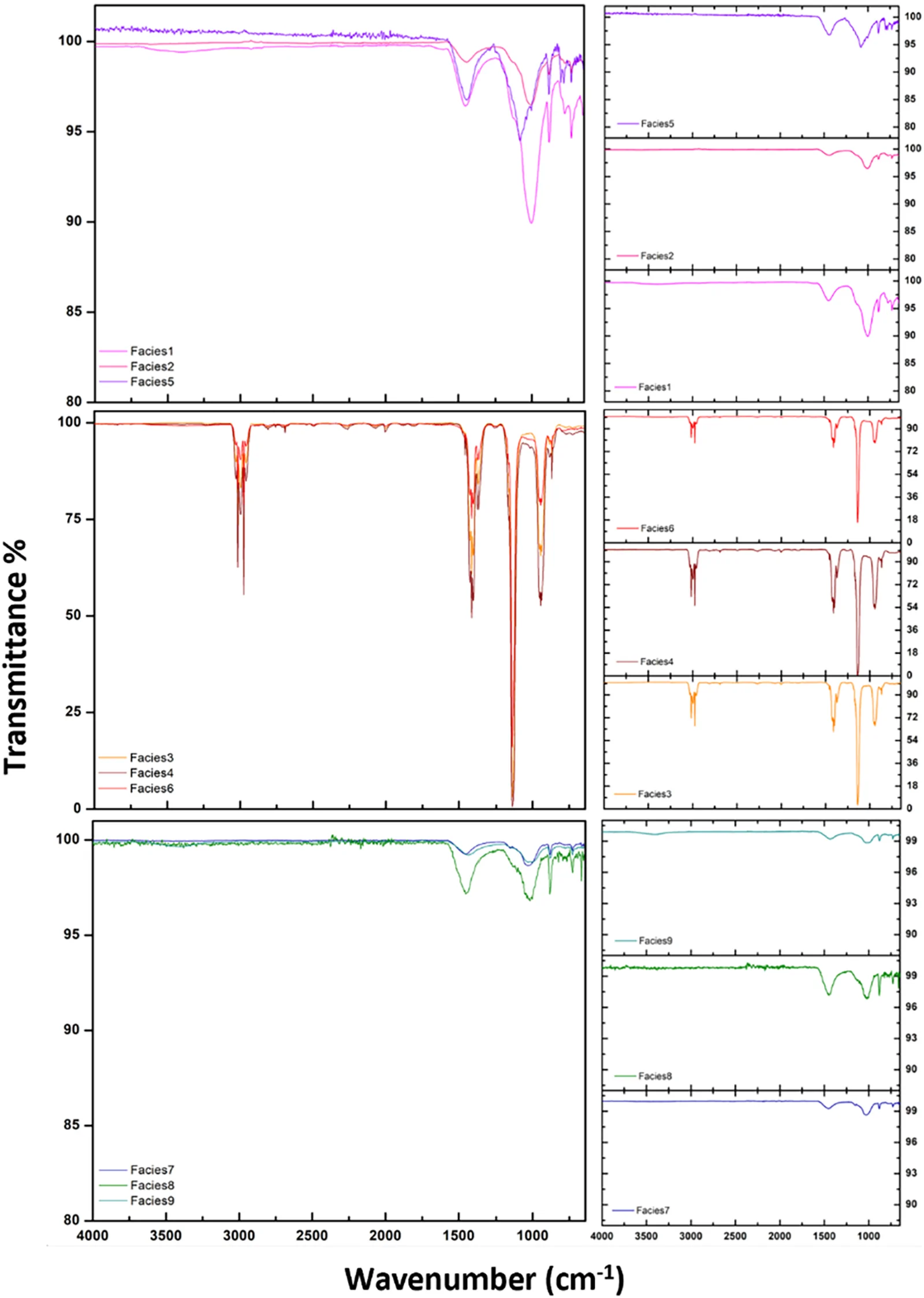
Fourier transform infrared (FTIR) spectra of all nine BD-139 rock facies grouped by mineralogical classification. Group 1 (Facies 3, 4, and 6) spectra display characteristic aromatic C–H stretching (∼3050 cm^–1^), aliphatic C–H stretching (∼2800–3000 cm^–1^) indicative of organic matter, and strong Si–O/Al–O asymmetric stretching vibrations (∼1000–1100 cm^–1^) consistent with a silicate-rich mineral matrix containing organic matter. Group 2 (Facies 1, 2, and 5) spectra exhibit a prominent carbonate band at ∼1450 cm^–1^ attributable to calcite/carbonate vibrations, with an absence of organic signatures. Group 3 (Facies 7, 8, and 9) spectra show no organic signatures and are dominated by silicate framework absorption, with the carbonate component indicated by thermogravimetry assessed through the diagnostic ν2 and ν4 carbonate bands. All spectra were acquired using a PerkinElmer Spectrum 3 FTIR spectrometer.

FTIR of Saline solution seen in Figure 4, stretches for O–H bond (∼3000–3600 cm^–1^) in both before the adsorption and after the adsorption analysis can be observed, and this is due to the presence of liquid water. The broad water peak is more pronounced before the adsorption analysis, and this signifies the granular pieces in the physical observation after the adsorption experiment was completed. This is also observed parallelly at the peak of 1650 cm^–1^, which indicates the loss of liquid water after the adsorption process. Low transmittance can be seen around 1350 cm^–1^ ∼38% before the experiment and that same peak shifted to more transmittance ∼58% at the after. This peak is associated with organic salts, and this shifting suggest in decrease of concentration of the salts or the reduction of the density of the salts after the adsorption.^20,21^ Between the before and after FTIR study, we can conclude that the chemical composition remains the same; however, the after sample shows a more depleted version of the before sample.

**Figure 4:**
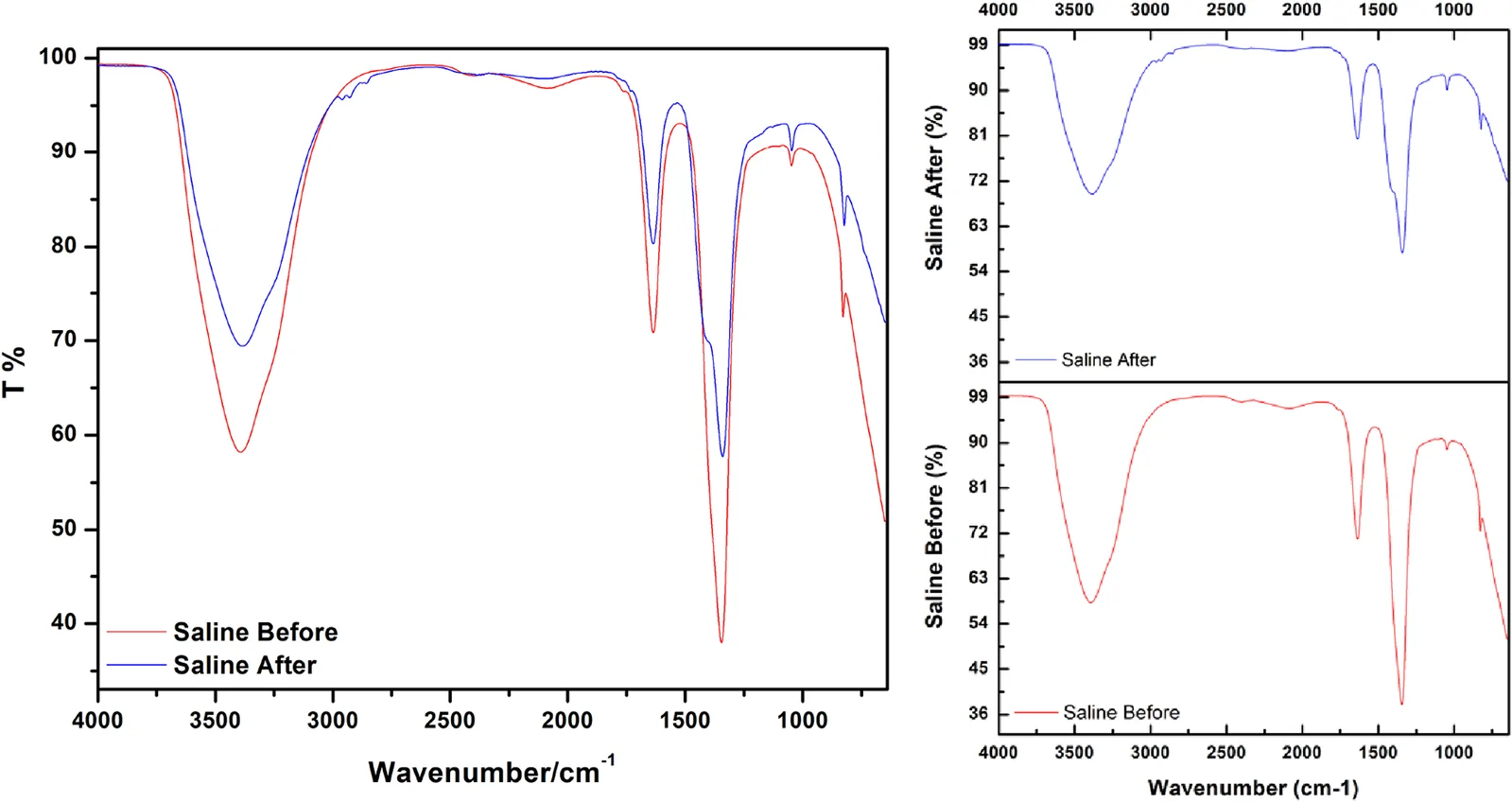
FTIR spectra of the synthetic brine solution before and after high-pressure CO_2_ absorption at 25 °C. The broad O–H stretching band (∼3000–3600 cm^–1^) and the H–O–H bending peak at ∼1650 cm^–1^ are both attenuated in the postabsorption spectrum, consistent with loss of liquid water and physical transformation of the solution to a coarse granular solid. A transmittance shift at ∼1350 cm^–1^ from ∼38% (before) to ∼58% (after) indicates a reduction in organic salt concentration or density, attributed to salt precipitation driven by CO_2_-induced acidification at elevated pressure. The overall spectral profile confirms that the chemical composition of the residual solid retains the ionic constituents of the original brine, albeit in a more depleted form.

### 3.3 Scanning Electron Microscopy (SEM) Analysis

The SEM analysis shows a similar physical trait conferred to the facies grouping. EDS mapping resolves the silicon (Si) distribution and thereby characterizes the matrix of each facies. Si in all of the EDS investigations is the second-most intense signal after oxygen, confirming a silicate-dominant matrix typical of aluminosilicate or quartz-bearing rocks. Beyond the dominant Si and O signals, the EDS spectra indicate minor elements such as Mg, Al, K, Ca, and Fe, suggesting the presence of aluminosilicate phases and trace accessory minerals. These traces may, to a certain level, influence the CO_2_ interaction potential in subsurface applications. Group 2, which consists of Facies 1, Facies 2, and Facies 5, reveals an aggregated microstructure with irregular grain morphologies and extensive textural heterogeneity. This leads to an indication of enhanced surface area and a possibility for a reactive material interface. Facies 3, Facies 4, and Facies 6 under the categorization of group 1 exhibit angular, crystalline particles with sharp edges. All of them are densely packed, with Facies 4 being the least dense one in comparison to Facies 3 and Facies 6. These features indicate a more uniform composition with reduced microporosity and coarser structure relative to group 1 facies. Morphologically, Group 3 resembles Group 2. Figure 5 shows the morphology for the 9 facies at the same level of magnification by SEM images. The detailed SEM-SE images and EDS maps for all facies are included in the electronic Supporting Information file (ESI) Figures S1 to S9.

**Figure 5:**
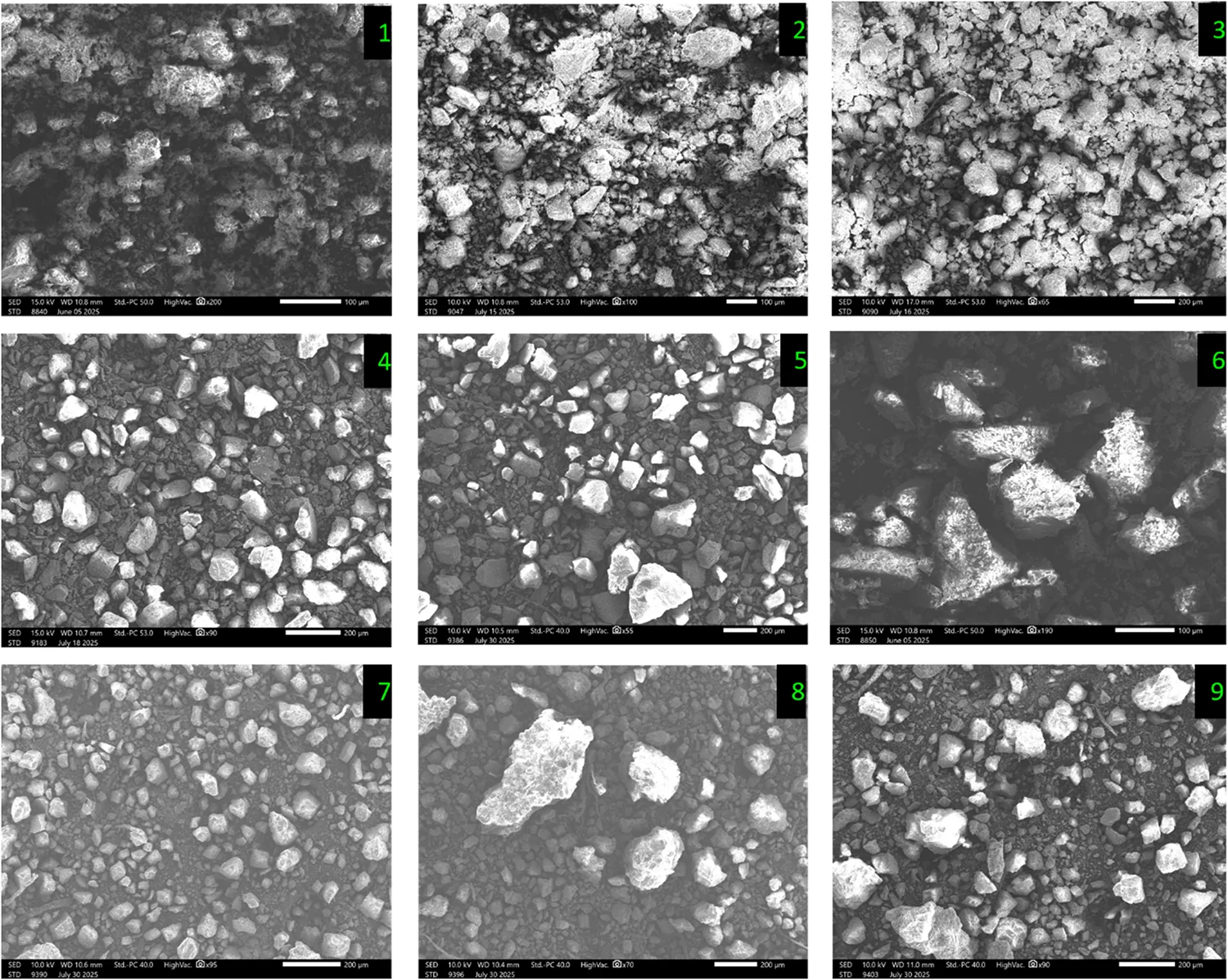
Representative scanning electron microscopy (SEM-SE) images of all nine BD-139 rock facies acquired at equivalent magnification using a JEOL IT-200 InTouch SEM at an accelerating voltage of 10–20 kV. Group 1 facies (Facies 3, 4, and 6) exhibit densely packed, angular crystalline particles with sharp edges and reduced microporosity, consistent with their silicate-dominant, low-surface-area composition. Group 2 (Facies 1, 2, and 5) and Group 3 (Facies 7, 8, and 9) facies display aggregated microstructures with irregular grain morphologies and pronounced textural heterogeneity, indicative of enhanced reactive surface interfaces. Energy-dispersive spectroscopy (EDS) confirms silicon (Si) and oxygen (O) as the dominant elemental signals across all groups, with trace contributions from Al, Mg, K, Ca, and Fe suggesting the presence of aluminosilicate phases and accessory minerals.

### 3.4 Brunauer–Emmett–Teller (BET) Surface Area Analysis

BET analysis characterizes any solid material based on an atomic level of unreactive gas adsorption to determine its surface area. This experiment was done with Liquid Nitrogen to drop the sample temperature to 77 K or −196.15 °C to avoid any reactivity between the rock sample and the N_2_ gas. Table 2 shows the final results for the BET experimentation. Facies 5 shows a data point 17.15 m^2^/g, the highest, which indicates a higher proportion of micropores. Group 1 facies represents the tightest material in the table lot which is less than 0.3 m^2^/g, indicating limited internal surface complexity.^22–24^ Isochoric assessment of the all of the rock samples with CO_2_ was done at 25 °C, 35 and 45 °C, a significantly higher temperature environment as opposed to N_2_ adsorption during the BET analysis, which was at −196.15 °C. The vast ambient difference between the two adsorptions indicates a vast difference in kinetic energy between the CO_2_ and N_2_ molecules. Kinetic Diameter measures as angstroms Å, stands as the smallest dimension a particular molecule presents during its thermal motion. This then transpires to the key aspect of understanding why the uptake values differ.^25,26^ The kinetic diameter of N_2_ is 3.64 Å (0.364 nm) and that of CO_2_ is 3.30 Å (0.330 nm). This value determines the ease with which a molecule passes through a porous material. While a difference of 0.34 Å (0.034 nm) appears negligible, it is significant at the subnanometre scale. CO_2_ can enter ultramicropores (pores below 0.7 nm on the IUPAC definition) that nitrogen cannot access.^22,27,28^ CO_2_ is a nonpolar molecule with no permanent dipole moment, but it carries a large permanent quadrupole moment (approximately −13.4 × 10^–40^ C m^2^), some three times that of N_2_ (approximately −4.7 × 10^–40^ C m^2^). The correspondingly stronger interaction between the CO_2_ quadrupole and the electric field gradients at polar mineral surfaces can produce a more densely packed adsorbed layer than is attainable with N_2_.^29^ This suggests that the facies sample with significant microporosity substantiates that the isochoric CO_2_ adsorption analysis will yield a higher surface area than the N_2_ adsorption in the BET study. With kinetic diameter and temperature difference, the diffusion between the N_2_ adsorption and CO_2_ implies that the BET surface area does not correlate with the measured uptake.^30^ Figures S10–S18 show the N_2_ adsorption data of the BET analysis for the rock samples.

**Table 2:** BET Surface Area Analysis Results for the Explored Rock Samples

facies – BD-139 sample	the highest single point surface area (m^2^/g)	the highest BET surface area (m^2^/g)
1	0.79	0.68
2	1.26	1.12
3	0.34	0.25
4	0.27	0.22
5		17.15
6	0.21	0.14
7	2.61	2.52
8	1.37	1.12
9	4.97	4.90

Two limitations of surface area determination should be noted. Nitrogen adsorption at 77 K approaches its practical lower limit below approximately 1 m^2^ g^–1^, where the dead-volume correction becomes comparable with the adsorbed quantity and krypton adsorption is the recommended alternative.^22^ The values reported for Facies 3, 4, and 6 (0.25, 0.22, and 0.14 m^2^ g^–1^) lie within this regime and carry correspondingly large relative uncertainty; they are used here as upper-limit estimates of the nitrogen-accessible surface. Second, nitrogen adsorption at 77 K does not characterize ultramicroporosity, for which CO_2_ adsorption at 273 K combined with nonlocal density functional theory analysis is the reference method;^22^ the ultramicropore contribution invoked below is therefore inferred rather than measured directly, and its direct determination is identified as a priority for further work.

The BET surface areas obtained in this study were compared with those reported for Marcellus shale by Abdulkareem et al.,^6^ who reported BET surface areas ranging from approximately 18 to 42 m^2^/g for their shale samples. In the present study, the BET surface areas ranged from 0.14 to 17.15 m^2^/g, with Facies 5 (17.15 m^2^/g) falling at the lower end of the range reported for the Marcellus shale, whereas most of the investigated facies exhibited substantially lower surface areas. This difference is not unexpected despite the low permeability of both rock types because permeability and BET surface area describe different aspects of the pore system. Permeability primarily reflects the connectivity and ability of fluids to flow through the pore network, whereas BET surface area reflects the amount of gas-accessible surface associated mainly with the micro- and mesopore structure. The relatively higher BET surface areas reported for the Marcellus shale may be associated with its distinct mineralogical and organic composition, including relatively high total organic carbon (3.6–17.2 wt %) and clay mineral contents reaching approximately 48–50 wt %, as well as its predominantly mesoporous pore structure with characteristic pore sizes of approximately 3–5 nm.^6^ In contrast, the investigated facies in this study represent a different lithological and mineralogical system, and their lower BET surface areas indicate a comparatively smaller gas-accessible internal surface area. Therefore, the fact that both formations can be classified as tight rocks does not necessarily imply comparable BET surface areas. The comparison highlights that BET surface area is controlled by pore size distribution, pore accessibility, mineralogy, and organic-matter content in addition to the overall permeability of the rock.

### 3.5 High-Pressure Capture of CO_2_ by a Isochoric System

#### 3.5.1 CO_2_ Adsorption on Facies-BD-139

The high-pressure isochoric CO_2_ adsorption profiles, evaluated across a pressure spectrum of 10–35 bar and isotherms of 25, 35, and 45 °C (Figure 6), reveal a distinct performance hierarchy among the geological facies. At the baseline isotherm of 25 °C (Figure 6a), Facies 3 and 4 exhibit comparable initial uptakes at 10 bar (116.8 mg/g and 117.2 mg/g, respectively); however, as fluid pressure increases to 35 bar, Facies 3 gives the highest apparent uptake, reaching 855.7 mg/g compared to 715.1 mg/g for Facies 4 (Figure 6b). Across all test conditions, Facies 3 consistently yields the highest apparent uptake among the nine facies.

**Figure 6:**
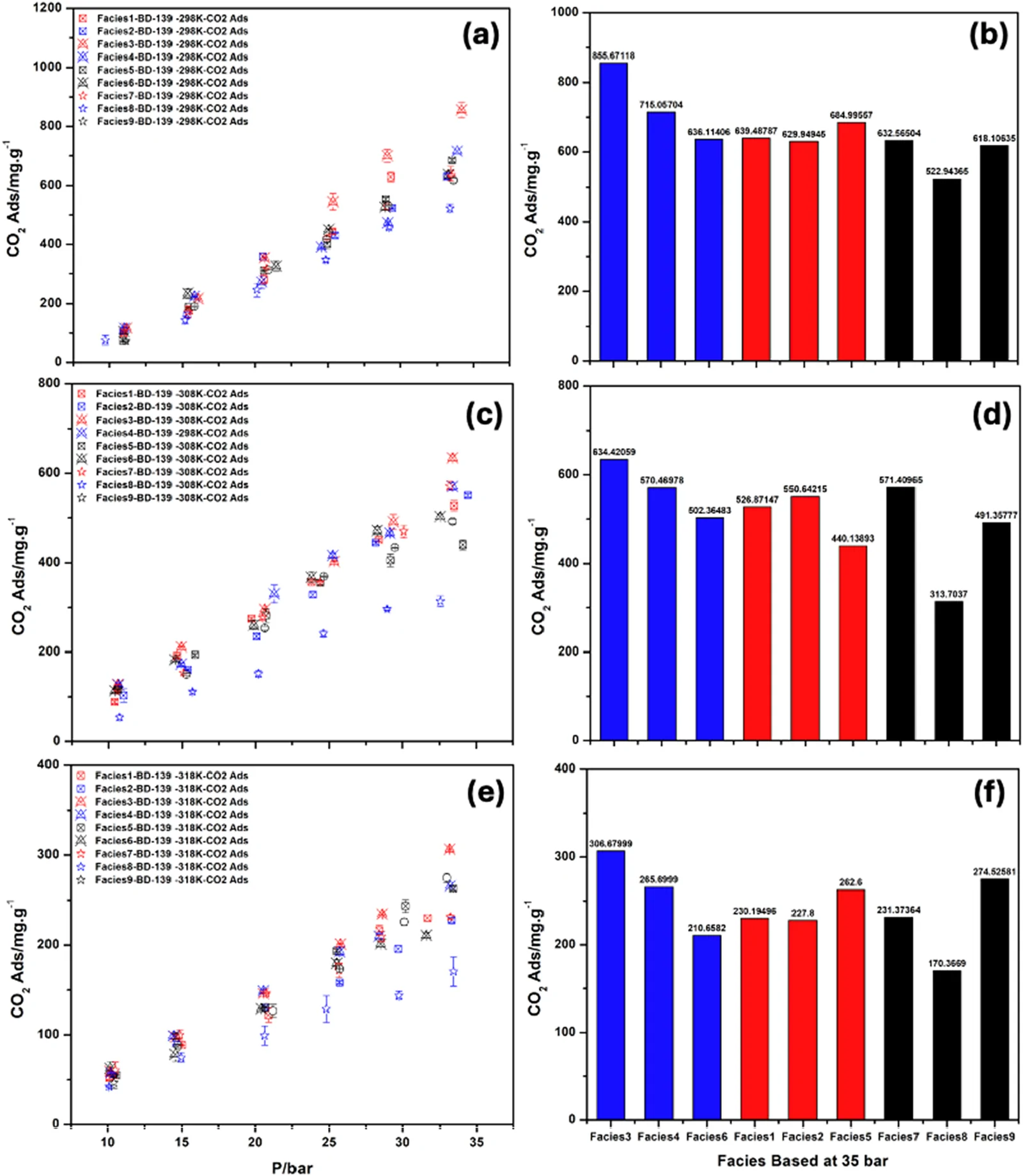
High-pressure isochoric CO_2_ adsorption profiles for nine BD-139 rock facies evaluated at 25 °C (a, b), 35 °C (c, d), and 45 °C (e, f). The left column (panels a, c, e) details the adsorption isotherms across a pressure range of 10–35 bar. The right column (panels b, d, f) isolates and compares the maximum adsorption capacities achieved at 35 bar for each corresponding temperature. Adsorption capacities are reported in mg/g.

Across all facies, the uptake increases monotonically with pressure and is suppressed at the highest temperature, as expected for an exothermic sorption process. Facies 3 consistently yields the highest apparent uptake under all conditions, reaching 855.7 mg/g at 25 °C and 35 bar. The preserved performance hierarchy among the facies groups across all three isotherms reinforces that mineralogical composition and surface chemistry dominate over kinetic effects in determining CO_2_ uptake.

The CO_2_ adsorption capacities obtained in the present study are comparatively higher than those reported in previous studies of shale and calcite-rich unconventional reservoirs. For example, Abdulkareem et al.^6^ and Samara et al.^8^ reported CO_2_ adsorption data for Marcellus and Jordanian unconventional reservoir rocks, respectively, with the reported adsorption quantities expressed on an excess-adsorption basis. In contrast, the present study reports the absolute CO_2_ adsorption. These two quantities are not directly equivalent. Excess adsorption represents the excess amount of CO_2_ associated with the adsorbed phase relative to the amount that would occupy the same adsorbed-phase volume at the bulk gas density, whereas absolute adsorption represents the total amount of CO_2_ associated with the adsorbed phase. The difference between the two quantities becomes increasingly important at elevated pressures, because the bulk CO_2_ density increases with pressure.

In the present study, the absolute amount of CO_2_ adsorbed was determined from the difference between the initial and final numbers of moles in the system using the effective free volume after subtracting the sample volume. The number of moles at each state was calculated using the real-gas equation (PV = *ZnRT*), with the compressibility factor (*Z*) obtained from REFPROP (NIST)^31^ at the corresponding experimental pressure and temperature. Therefore, the reported adsorption capacity does not rely on an ideal gas approximation. The substantially higher values observed in the present study should consequently be interpreted with consideration of the different adsorption definitions in addition to differences in rock type, mineralogical and organic composition, pore structure, accessible surface area, sample preparation, pressure and temperature, and experimental methodology. In particular, the use of powdered samples and the determination of absolute rather than excess adsorption can contribute to higher reported uptake values compared to studies reporting excess adsorption on differently prepared rock samples. Nevertheless, these factors should be considered collectively rather than being attributed to a single parameter.

The high apparent uptake of Facies 3, and more broadly of the Group 1 specimens, must be contextualized alongside their physicochemical characterization. Despite exhibiting the lowest N_2_-accessible macroscopic surface areas (<0.3 m^2^/g) during BET analysis, these silicate-rich, organic-bearing matrices provide a highly favorable environment for CO_2_ retention. This apparent discrepancy highlights the critical role of ultramicroporosity (pores below 0.7 nm) within the rock architecture. This behavior may also be associated with nanoscale confinement effects. Within ultramicropores, the interaction between CO_2_ molecules and the pore walls becomes increasingly significant, potentially promoting enhanced CO_2_ accumulation and pore-filling behavior compared with adsorption on more open surfaces. Consequently, CO_2_ uptake does not necessarily scale directly with the N_2_–BET surface area measured at 77 K, particularly when a portion of the pore network is accessible to CO_2_ under the adsorption conditions but is poorly represented by N_2_ adsorption. Such confinement effects are capable of decoupling CO_2_ uptake from the nitrogen-accessible surface area measured at 77 K, although, as established in Section 3.5.3, they do not account for the magnitudes reported here. However, nanoscale confinement should not be interpreted as implying unrealistically high CO_2_ densities within the pore space; even under strong confinement and pore-filling conditions, the confined CO_2_ density is expected to remain within a physically reasonable range relative to the density of liquid CO_2_ under comparable conditions. While the cryogenic conditions of N_2_ BET analysis restrict gas diffusion into these tight networks, the slightly smaller kinetic diameter of CO_2_ (3.30 Å vs 3.64 Å for N_2_) coupled with the higher thermal kinetic energy at ambient temperatures permits deep penetration into the solid framework.

Furthermore, the strong quadrupole moment of the CO_2_ molecule likely induces favorable electrostatic interactions with the polar domains of the silicate matrix and the organic functional groups identified via FTIR, promoting dense molecular packing at the solid–gas interface that N_2_ molecules cannot replicate. The isochoric profiles also underscore the classical thermodynamic dependencies of physisorptive systems. Across all nine facies, CO_2_ uptake scales monotonically with pressure (Figure 6a,6c,6e). This behavior is consistent with concentration-driven diffusion; as the system pressure increases, the governing effect of temperature on the adsorption rate diminishes, driving the progressive occupation of available active surface sites.^14^ Conversely, the system exhibits strong temperature attenuation when comparing the maximum capacities across discrete isotherms (Figure 6b,6d,6f). For Facies 3, the uptake at 10 bar is 116.8 mg/g at 25 °C and 127.9 mg/g at 35 °C, values that are equal within the combined measurement uncertainty derived in Section 2.4, whereas the reduction to 59.5 mg/g at 45 °C under the same pressure is significant. This capacity degradation at elevated isotherms is an expected manifestation of Le Chatelier’s principle for exothermic adsorption processes; as the thermal kinetic energy of the adsorbed phase exceeds the depth of the local potential energy well at the material surface, desorption is favored, resulting in inherently lower capacities at higher temperatures.^32^ Consequently, while fluid pressure serves as the primary kinetic driver for pore saturation, the intrinsic mineralogical chemistry dictates the absolute upper limit of sequestration capacity under specific geothermal gradients. Figure 7 shows a heat map for the temperature and pressure effect on the adsorption capacity by Facies 3, as it gives the highest apparent uptake among the facies examined.

**Figure 7:**
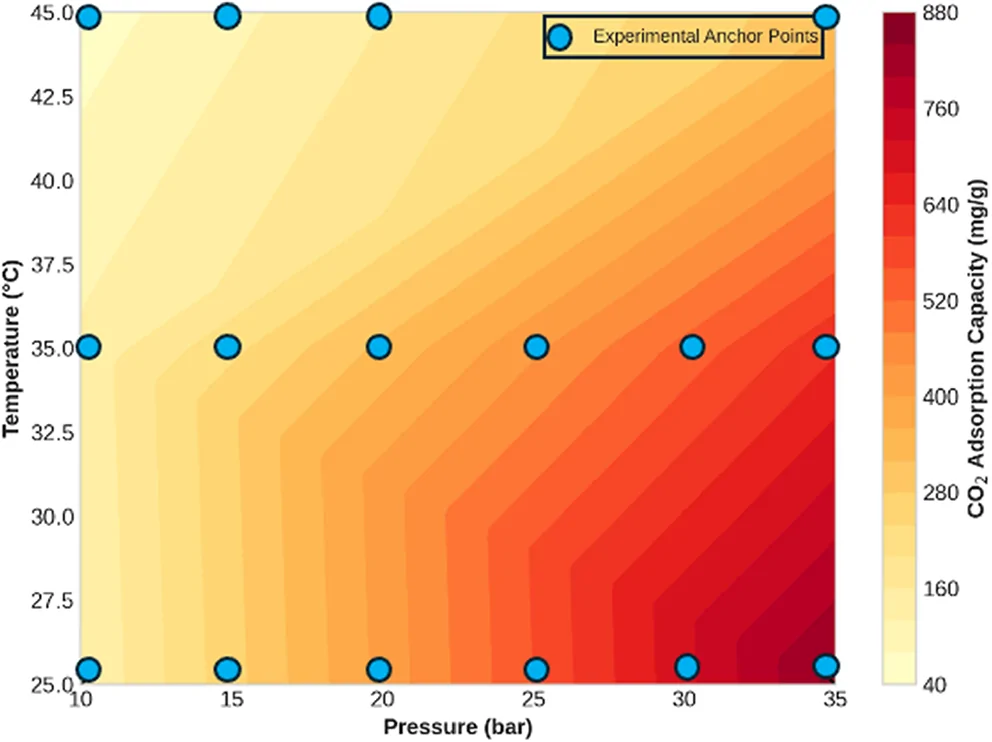
Contour map of the apparent isochoric CO_2_ uptake (mg/g) for Facies 3 as a function of pressure and temperature. The gradient indicates the pressure and temperature dependence of the uptake. The values plotted are apparent isochoric uptake as defined in Section 3.5.3 and are not storage capacities.

Regarding the implications of the measured adsorption capacity for in situ CO_2_ storage, the investigated facies exhibit substantial differences in porosity, permeability, and surface characteristics. The relatively higher-porosity and higher-permeability intervals, particularly Facies 3 and Facies 5, provide greater potential for volumetric pore-space storage because of their larger pore volume and improved fluid accessibility. Facies 3, which has a porosity of 10–20% and permeability of 74.95 mD, accounts for approximately 19% of the core and exhibited the highest measured CO_2_ adsorption capacity, reaching 855.7 mg/g at 298 K and 35 bar. Facies 5 also has a porosity of 10–20% and permeability of 8.29 mD and represents approximately 14% of the core. In contrast, the low-permeability facies, particularly Facies 7 and 8 with permeabilities below 0.4 mD, have limited fluid accessibility and contribute less to effective pore-volume storage, although their low permeability may favor longer-term containment by restricting fluid migration.

The BET surface areas ranged from 0.14 to 17.15 m^2^/g. Interestingly, facies 3 exhibited a relatively low BET surface area of 0.25 m^2^/g despite its high apparent CO_2_ uptake, whereas facies 5 exhibited the highest BET surface area of 17.15 m^2^/g. This indicates that CO_2_ adsorption is not controlled solely by the total BET surface area, including the increase in exposed surface area resulting from the size reduction of the intact rock to a ground sample/powder, but it is also influenced by pore structure, mineralogical composition, surface chemistry, micropore accessibility, and the affinity of specific adsorption sites. Although grinding the samples increases the exposed surface area and may provide access to surfaces that are less accessible in intact rock, the observed adsorption behavior does not indicate that the increase in the BET surface area associated with pulverization alone was the dominant factor controlling CO_2_ uptake. In particular, the high adsorption capacity of facies 3, despite its relatively low BET surface area, demonstrates that the adsorption response cannot be attributed simply to the increase in surface area produced by grinding. Nevertheless, the BET and CO_2_ adsorption measurements were performed on powdered samples to increase surface accessibility and facilitate equilibration within practical laboratory time scales. Therefore, the measured adsorption capacities should be interpreted as intrinsic adsorption potentials of the prepared rock matrix rather than direct estimates of the adsorption capacity of the intact reservoir rock.

At the reservoir scale, the effective CO_2_ storage capacity will depend on the combination of available pore volume, rock volume and density, permeability and pore connectivity, and the fraction of the measured surface area that is accessible to CO_2_ under in situ conditions. Thus, the high adsorption capacity measured for a powdered sample cannot be directly converted to a field-scale storage capacity without accounting for the accessibility of the adsorption surface and the transport limitations imposed by an intact pore network. This limitation is particularly important for the low-porosity and low-permeability facies, where restricted matrix transport and limited pore connectivity may substantially reduce the rate and extent of CO_2_ penetration into the rock matrix. Accordingly, the adsorption results provide a measure of the intrinsic sorption potential of each facies, while the actual in situ storage contribution will be governed by sorption capacity, accessible pore volume, and transport properties.

#### 3.5.2 CO_2_ Absorption by Saline Solution and Implications for Mineral Trapping

The CO_2_ absorption profiles for the synthetic saline solution, evaluated across 25 and 35 °C isotherms (Figure 8), introduce a critical geochemical contrast to the dry rock facies adsorption data. At 25 bar, the brine system achieves absorption capacities of 77.5 mg/g at 25 °C and 81.78 mg/g at 35 °C. These two values differ by approximately 5% and are equal within the combined measurement uncertainty, so no temperature dependence is claimed over this interval; the physical solubility of CO_2_ in aqueous media decreases by roughly 15% between 25 and 35 °C at fixed pressure, and a departure from that behavior would require a larger and better resolved difference than is observed here. The absolute values are also higher than the accepted solubility of CO_2_ in pure water at 25 bar and 298 K, approximately 0.7 mol kg^–1^ or 31 mg g^–1^, which salting-out in a concentrated multivalent brine would reduce further; the same interpretive limits set out in Section 3.5.3 for the rock specimens therefore apply to the brine measurements, which are reported as apparent isochoric uptake. What the brine experiments establish unambiguously, independently of the magnitude of the uptake, is that the system undergoes a reactive transformation under CO_2_ at pressure.

**Figure 8:**
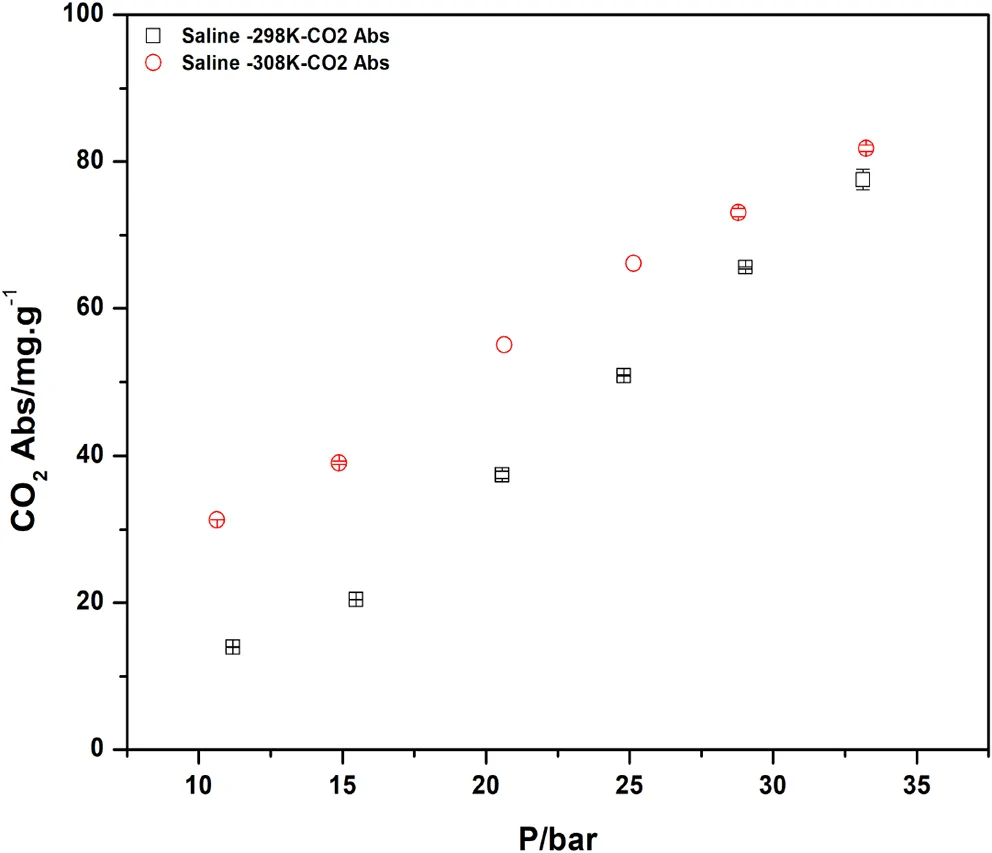
CO_2_ absorption capacity of the synthetic brine solution as a function of pressure at two temperature isotherms (25 and 35 °C), reported in mg/g. Absorption values of 77.5 mg/g at 25 °C and 81.78 mg/g at 35 °C were measured at 25 bar. The values obtained at 25 and 35 °C are equal within the combined measurement uncertainty, and no temperature dependence is claimed over this interval. The postexperiment transition of the brine from an aqueous solution to a coarse granular solid is consistent with CO_2_-induced acidification and mineral trapping via carbonate and oxide precipitation, supporting the role of ionic brine environments in enhancing long-term geological CO_2_ permanency.

The mechanistic basis for this enhanced uptake is corroborated by a distinct postexperiment phase transition: the saline matrix evolved from an aqueous solution into a dense, coarse granular solid. Upon dissolution under high pressure, CO_2_ forms carbonic acid, rapidly lowering the solution’s pH. In a high-salinity environment rich in divalent and trivalent cations, this acidification shifts the local thermodynamic equilibrium, triggering the supersaturation and massive precipitation of carbonate and oxide phases, specifically those incorporating Ca^2+^, Mg^2+^, and Fe^3+^. This precipitation continuously depletes the aqueous phase of dissolved carbon species, acting as a chemical sink that drives the further dissolution of CO_2_ from the gas phase to maintain equilibrium.

The evidence for this pathway presented here is the phase transition itself, together with the depletion of the dissolved ionic constituents indicated by the FTIR comparison of Figure 4. Mineralogical identification of the recovered solid by powder X-ray diffraction and SEM-EDS, which would establish the precipitated phases and distinguish carbonate phases formed in response to CO_2_ dissolution from ferric oxyhydroxide formed by hydrolysis of the Fe­(III) constituent of the synthetic brine, was not performed in this study and is identified as the next step in establishing the mineral trapping pathway.

Integrating these aqueous dynamics with the high-pressure rock adsorption data is fundamental to accurate reservoir-scale evaluation. In true subsurface environments, the BD-139 facies do not exist as isolated, dry matrices; their porous networks are deeply saturated with connate brine. The synthetic brine absorption experiments directly simulate interstitial reactivity. When the injected CO_2_ interfaces with the brine-saturated rock, the connate fluid acts as a reactive mediator. It facilitates the dissolution of native rock minerals, particularly in the heterogeneous, carbonate-bearing group 2 facies, and provides the ionic transport network necessary for secondary mineral precipitation. Consequently, while the intrinsic rock matrix provides the primary surface area for physical storage (adsorption trapping), interstitial brine facilitates direct chemical conversion (mineral trapping). This synergistic mechanism permanently locks the CO_2_ into stable solid phases within the pore space, significantly enhancing the long-term geological security and permanence of Michigan Basin storage sites.

#### 3.5.3 Physical Bounds on the Measured Uptake

The magnitudes reported above require explicit examination against the pore volume and surface area measured for the same specimens. For facies 3, the measured nitrogen-accessible surface area of 0.25 m^2^ g^–1^ corresponds to a complete CO_2_ monolayer of approximately 0.10 mg g^–1^, taking a molecular cross-sectional area of 0.19 nm^2^. Taking the upper bound of the visual porosity of this facies (20%) together with a grain density of 2.65 g cm^–3^ gives a pore volume of 0.094 cm^3^ g^–1^. Occupying that volume with CO_2_ at its bulk density at 35 bar and 298 K (71 kg m^–3^) corresponds to 6.7 mg g^–1^; occupying it in its entirety with liquid CO_2_ at 0.71 g cm^–3^ corresponds to 67 mg g^–1^; and filling it at the van der Waals adsorbed-phase density of approximately 1030 kg m^–3^, conventionally applied to CO_2_ in rock,^33^ corresponds to 97 mg g^–1^. The last of these constitutes an upper bound on the CO_2_ that an intact specimen of this facies can hold under any mechanism, whether at the mineral surface or within the pore volume, and it is exceeded by the values reported in Section 3.5.1.

The origin of this discrepancy lies in the resolution of the isochoric method at the sample masses employed, and is quantified by the uncertainty analysis of Section 2.4. The sorbed amount is obtained as the difference between two mole numbers that are large relative to it: at 35 bar and 298 K, the 100 mL cell contains 174 mmol of CO_2_, against which the quantity determined for a 0.25 g specimen represents 2.8%. The relative uncertainty of the difference is therefore amplified by the factor n/Δ*n* ≈ 35, and the absolute resolution limit of the configuration is approximately 85 mg g^–1^. This limit is of the same order as the volumetric capacity of the specimens themselves (67–97 mg g^–1^ for the most porous facies), and for the majority of the facies, whose surface areas lie between 0.14 and 4.9 m^2^ g^–1^, it exceeds any capacity they can physically possess. The configuration used here is consequently unable to resolve a sorption capacity for these materials, and the quantities reported in Figures 6 and 7 are to be read as apparent isochoric uptake constituting an upper bound on the sorbed amount, not as a determination of an adsorption capacity. The same apparatus has been applied previously to CO_2_ absorption in liquid systems,^13,14,16^ in which sample masses are 2 orders of magnitude greater and the sorbed fraction of the cell inventory correspondingly larger; the limitation identified here is specific to the low-capacity, small-mass solid configuration rather than to the method itself.

Two further contributions, both smaller, are noted for completeness. The specimens are pulverized, which exposes the surface that is inaccessible in the intact rock and removes the transport limitations imposed by its pore network, so that any capacity measured on this material is an intrinsic property of the prepared matrix rather than of the formation. The reported quantity is also absolute rather than excess; although this distinction is important in principle when comparing with the excess quantities reported elsewhere,^6,8^ at 35 bar and 298 K a bulk gas density of 71 kg m^–3^ acting on a pore volume of order 0.09 cm^3^ g^–1^ makes the difference between the two definitions approximately 6–7 mg g^–1^, which is small relative to the values reported and cannot account for the discrepancy. Accordingly, the sorption data are not converted to storage capacities at any scale in this work, and the facies classification developed here rests on the thermophysical, mineralogical, and morphological characterization of Sections 3.1 to 3.4. Determination of the sorption capacities of these facies requires a configuration matched to their capacity range, either a substantially reduced ratio of void volume to sample mass or a gravimetric method with buoyancy correction, and this is identified as the principal objective for subsequent work.

A related consideration governs the transfer of these measurements to reservoir conditions. At the sampled interval, the formation pressure of approximately 110 bar exceeds the critical pressure of CO_2_ (73.8 bar), while the formation temperature of 34–37 °C lies immediately above the critical temperature (31.0 °C). CO_2_ in this formation is therefore in the dense phase at approximately 630–750 kg m^–3^, and the reservoir sits in the region in which the density of CO_2_ is most sensitive to small changes in pressure and temperature: a decline of a few tens of bar, or a temperature excursion of a few degrees, produces a disproportionate change in density and hence in the volumetric storage efficiency of the pore space. Two consequences follow for the present work. First, the distinction between excess and absolute sorbed quantities, which amounts to only 6–7 mg g^–1^ at the subcritical laboratory conditions used here, becomes an order of magnitude larger at in situ density and cannot be neglected in any reservoir-scale evaluation; laboratory measurements made below the critical point therefore cannot be transferred directly to formation conditions. Second, at 3200–3700 ft, this interval lies near the shallow limit at which CO_2_ remains dense, so that the sensitivity of stored density to pressure decline is itself a storage efficiency and containment consideration for this particular target. Sorption measurements spanning 100–125 bar at 30–37 °C, which bracket the critical point in both variables, are consequently required before the facies ranking established here can be expressed as a storage capacity.

### 3.6 Heat of Sorption of CO_2_ Storage

Heat of adsorption (λ) is an aspect that needs to be considered parallelly with the competence of the adsorption value. In thermodynamics, this is computed through the Clausius–Clapeyron equation by selecting the amount of CO_2_ adsorbed by a one particular facies at two isotherms. This gives an insight on the thermal effect on the CO_2_ molecule while being experimented with the Facies specimen.^14^ Furthermore, we can understand the kinetics behavior during the adsorption process and subsequently, the stability of this CO_2_ molecule once it is sequestered by the Facies for the complete assessment. Figure 9 shows the heat of adsorption for the Facies sample investigated in this work.

**Figure 9:**
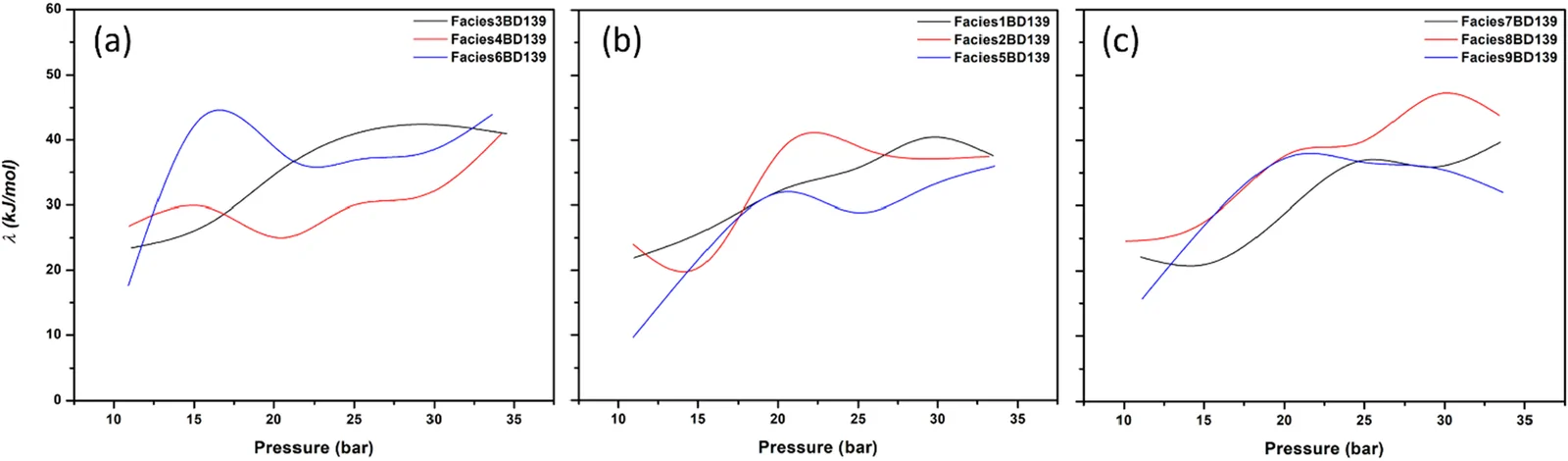
Heat of CO_2_ sorption (λ, kJ mol^–1^) for all nine BD-139 rock facies evaluated at constant pressure from paired isotherm data using the Clausius–Clapeyron relation. The isosteric heat, evaluated at constant surface loading, is presented in Figure 10. Results are presented by group classification. (a) Group 1: Facies 3, 4, 6; (b) Group 2: Facies 1, 2, 5; and (c) Group 3: Facies 7, 8, 9. All facies exhibit λ values below 60 kJ mol^–1^, indicating that CO_2_ interaction with all rock specimens proceeds predominantly via physical adsorption. At 30 bar, Facies 8 records the highest λ value of 47.22 kJ mol^–1^, followed by Facies 3 at 42.40 kJ mol^–1^, indicating greater resistance to CO_2_ desorption in these specimens.

Here, the value of enthalpy is computed in kilo joule heat per mole of the CO_2_ adsorbed (kJ/mol). All Facies specimens show heat of adsorption in the range of physisorption, which is less than 60 kJ/mol. The boundary between physical and chemical adsorption is not sharp, conventionally spanning approximately 40–80 kJ mol^–1^, and the highest value obtained here (47.22 kJ mol^–1^) lies within that transition range; the interaction is therefore described as predominantly, rather than exclusively, physical. Throughout this work, λ is reported as a positive quantity for an exothermic process, that is λ = −Δ*H*. Additionally, this heat of adsorption represents stability for its numerical value. i.e., the higher the λ value, the more strongly the CO_2_ molecule is retained by the facies sample. Here, Facies 3, being the promising choice for its highest value of adsorption in all three isotherms, can be challenged in their heat of adsorption values. From Group 1, Facies 3 has a calculated λ value of 42.40 kJ/mol CO_2_ at 30 bar. This value is the second-highest calculated λ value among all of the facies at 30 bar. Facies 8 holds the highest value at 47.22 kJ/mol. This value indicates stronger retention than that of Facies 3. However, this immovability factor can be debated if the end target is focused on the capability performance of the physical adsorption as opposed to the energy it requires to remove the bond once integrated. The heat of adsorption values comparison of the highest capacity facies of the three groups of the facies (3, 5, and 7) can be seen in Figure 10.

**Figure 10:**
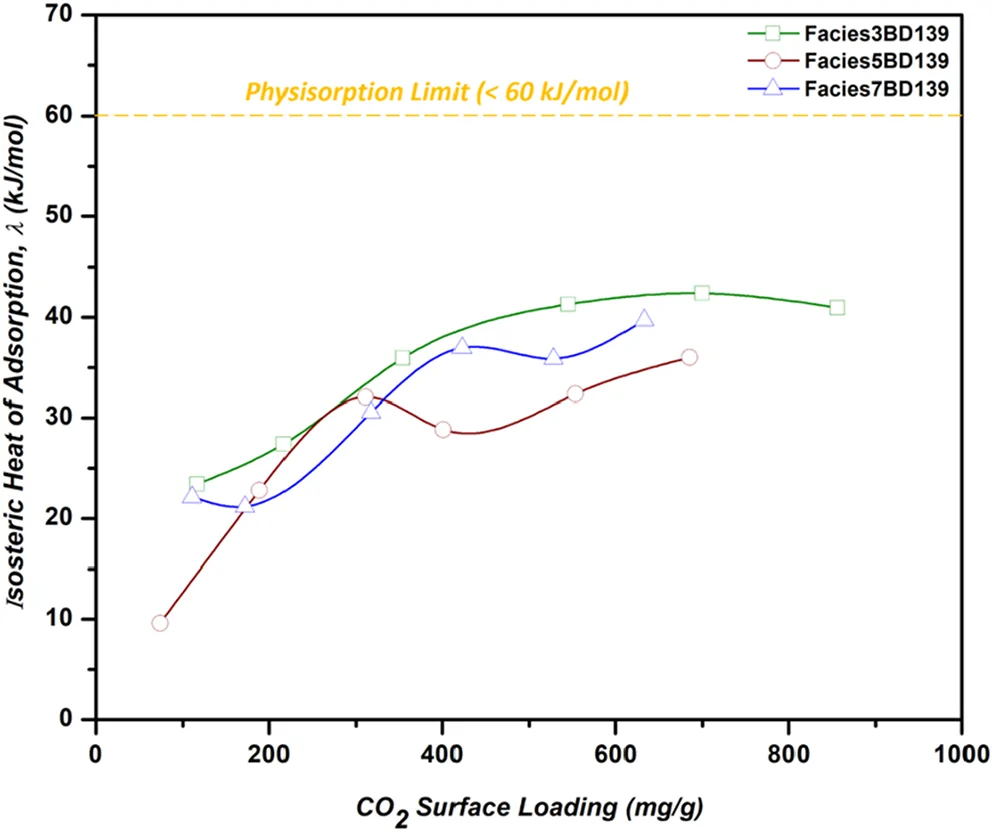
Thermodynamic Stability Profile. Isosteric heat of adsorption plotted as a function of CO_2_ surface loading for representative facies across the three mineralogical classifications (Facies 3, 5, and 7). All interaction energies remain strictly below the 60 kJ mol^–1^ threshold across the saturation curve, confirming that physical adsorption governs CO_2_ trapping at all stages of pore filling.

The heat of absorption for the saline solution was calculated as −69.60 kJ/mol at 10 bar and 3.94 kJ/mol at 35 bar. Reported on the same convention used for the rock specimens, the value obtained at 10 bar corresponds to a strongly exothermic process. It is considerably more exothermic than the enthalpy of physical dissolution of CO_2_ in aqueous media, approximately −20 kJ mol^–1^, and is therefore not attributable to dissolution alone; it is consistent with a combined contribution from dissolution and from the exothermic precipitation reactions evidenced by the solid recovered after depressurization. Values derived from only two isotherms separated by 10 K carry a large propagated uncertainty and are reported as indicative rather than as thermodynamic determinations. Because the cell is sealed and thermostated throughout, the granular character of the recovered material cannot be attributed to evaporative loss of water, which would remain within the closed volume; it reflects the precipitation described in Section 3.5.2. Figure 11 shows the absorption data of CO_2_ gas by brine solution.

**Figure 11:**
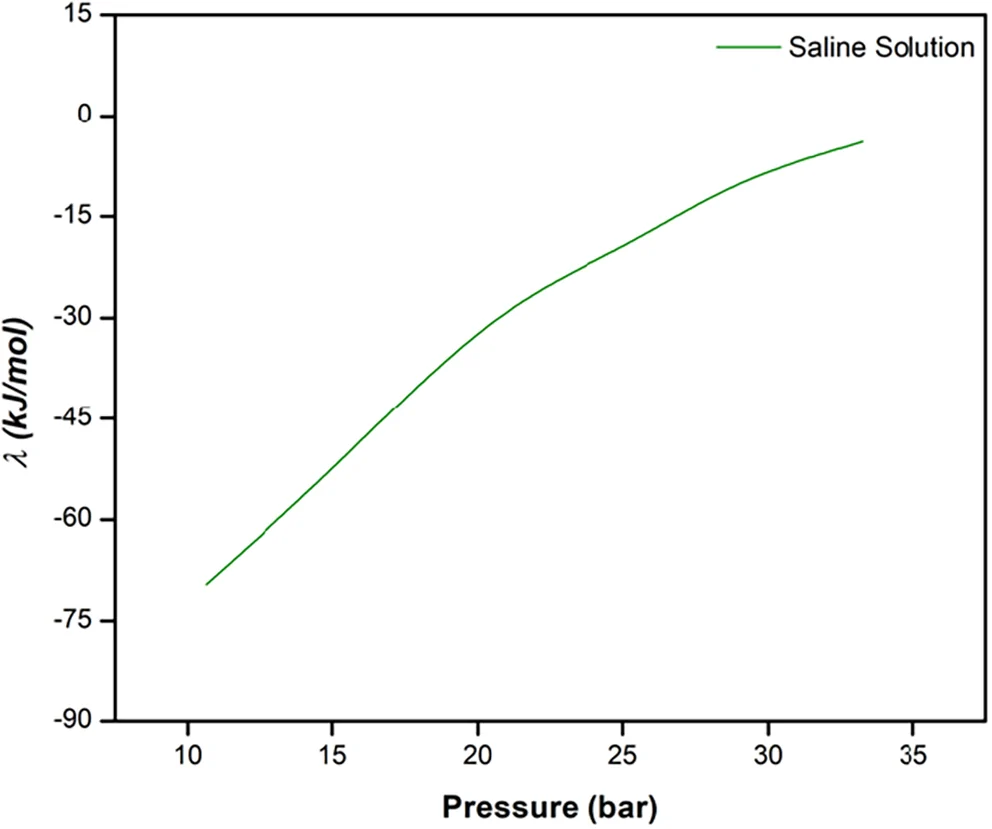
Heat of absorption for the synthetic brine was calculated as −69.60 kJ mol^–1^ at 10 bar and 3.94 kJ mol^–1^ at 35 bar, with the negative value at low pressure confirming the exothermic character of CO_2_–brine interactions.

The delineated multivariate relationship in Figure 12 shows a noticeable decoupling between the apparent CO_2_ uptake and the macroscopic N_2_–BET surface area within the investigation of the 9 Facies samples. These results challenge the traditional reliance of carbon sequestration on the surface area metrics as a primary predictor of CO_2_ performance. Facies 5 exhibited the highest expansive N_2_–BET surface area (the biggest bubble); however, it yielded an approximate uptake of CO_2_ of 685 mg/g. Here, a limitation was dictated by the weak thermodynamic affinity (λ ≈ 36.5 kJ/mol) of the sample. On the contrary, Facies F3 could achieve the highest maximum CO_2_ uptake of ∼855 mg/g, regardless of the possession of a smaller N_2_–BET surface area. The higher apparent uptake of this sample is associated with its elevated heat of sorption (λ ≈ 42.5 kJ/mol), emphasizing that surface affinity toward CO_2_ gas and the electrostatic interactions outweigh the bulk geometric surface area under these thermodynamic conditions. This discrepancy is essentially rooted in original mineralogical and structural heterogeneities of the facies, exclusively relating to the presence of ultramicroporosity (less than 0.7 nm), which remains undetectable by the standard N_2_-based characterization. Although cryogenic N_2_ sorption at 77 K is kinetically restricted from probing subnanometre pore networks, the narrow pore openings are largely available for CO_2_ molecules at the temperature of 25 °C because of its small kinetic diameter in addition to the strong quadrupole interactions with the polar surfaces of the minerals. Nevertheless, the results exhibited a critical diminishing-returns threshold. Facies 8 recorded the largest binding energy with CO_2_ molecules (λ ≈ 47.3 kJ/mol); however, it experiences the minimal overall adsorption capacity of ∼523 mg/g. This is an indication of the excessively elevated localized affinities capable of correlating with narrow, premature site saturation or volume-restricted pore configurations, proving that to maximize the sequestration of CO_2_ gas needs a harmonized structural regime adjusting both moderate-to-high adsorption enthalpy (40 to 43 kJ/mol) and reachable ultramicropore volume.

**Figure 12:**
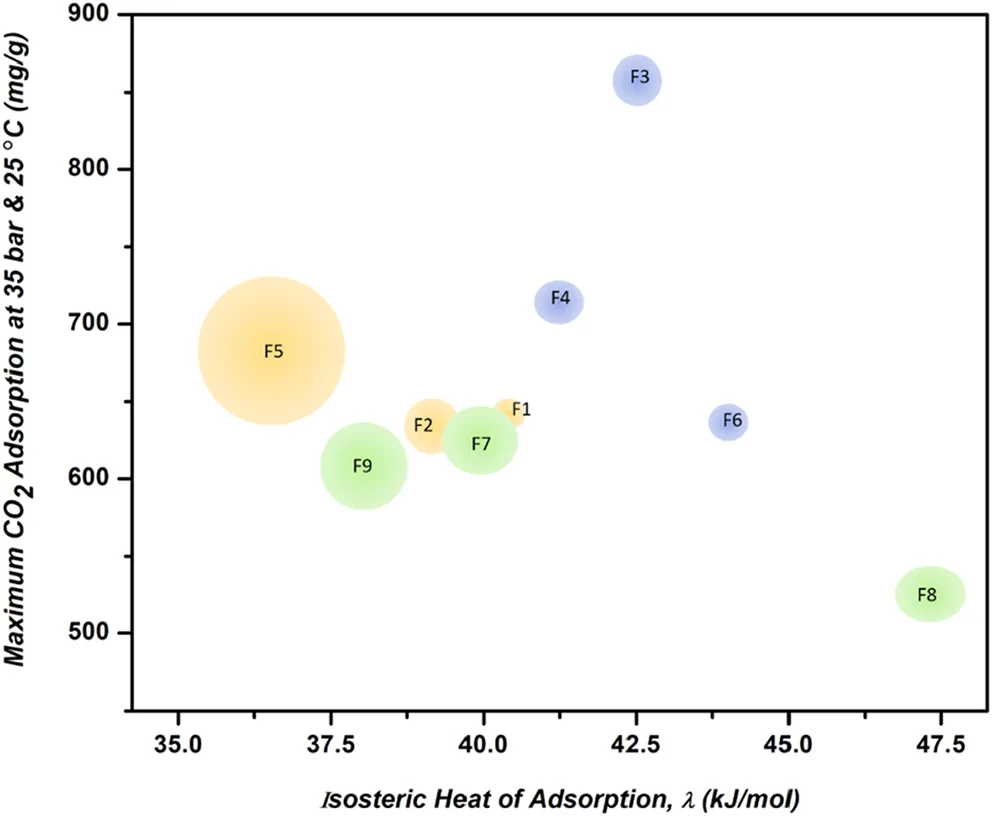
BET discrepancy analysis. Multivariable mapping of the apparent CO_2_ uptake for the BD-139 facie**s** at 35 bar and 25 °C. The plot correlates the isosteric heat of adsorption against maximum CO_2_ uptake, categorized by mineralogical group. Bubble diameter is proportional to the N_2_ BET surface area, visually demonstrating the decoupling of macroscopic surface area from the apparent CO_2_ uptake. The bounds governing the interpretation of these values are set out in Section 3.5.3.

### 3.7 Kinetics of CO_2_ Sorption

The kinetics behavior for a portion of the facies samples was analyzed. The kinetic profiles are presented as normalized CO_2_ uptake, calculated as *q*_t_/*q*_eq_ × 100, where *qt* represents the CO_2_ uptake at a given time and *q*_eq_ represents the equilibrium uptake determined at the end of the 1 h experiment. Thus, values exceeding 100% represent a transient uptake above the final equilibrium value rather than the equilibrium adsorption capacity. The initial overshoot reflects the rapid CO_2_ uptake during the early stage of the process, driven by the concentration gradient between the gas phase and the initially unsaturated adsorption sites. As the system approaches equilibrium, the initially accumulated CO_2_ may undergo redistribution within the pore network and between adsorption sites with different interaction energies, with partial desorption contributing to the subsequent decrease toward the equilibrium value. The overshoot is therefore considered a transient nonequilibrium phenomenon. Pressure and temperature were continuously recorded throughout the experiments and were incorporated into the calculation of the instantaneous CO_2_ uptake; however, the normalized presentation is used here to focus on the temporal evolution of the adsorption process and the time required to reach equilibrium.

A part of the transient excess may additionally reflect the finite response time of the temperature sensor relative to that of the pressure transducer during the charging step, as discussed in Sections 2.3 and 3.5.3. The normalized profiles are therefore used to compare the characteristic equilibration times of the facies with one another rather than to quantify the transient uptake itself. The corresponding pressure and temperature records for each experiment are provided in Figures S21–S23.

Facies 3, Facies 5, and Facies 7 were selected due to the incentive that each of them provided the highest adsorption values in their group cataloguing. The experimental adsorption rates for the selected facies were assessed at 10 and 35 bar for the isotherms at 25 °C, and this is shown in Figure 13. This gives an insight on the completion speed of the process of adsorption in the cell.^14^ Facies 3 shows that the maximum adsorption of CO_2_ at 10 bar was reached after 25 min. The time taken for completion of the adsorption process at 35 bar was faster at 15 min. Facies 5 shows a faster completion than Facies 3 for 10 bar, which is shown to be 21 and 15 min for 35 bar. Facies 7 shows a faster completion for 10 bar, which is 16 min. The uptake at 10 bar is slower and stepped in character. On the other hand, at 35 bar, the uptake curves for all three facies are faster and smoother. The pressure here is the driving factor for the completion of the adsorption process. The higher the pressure, the lesser is the time required for the experiment to reach equilibrium. The kinetic behavior of these facies defines the time required for the sorption process to reach equilibrium.

**Figure 13:**
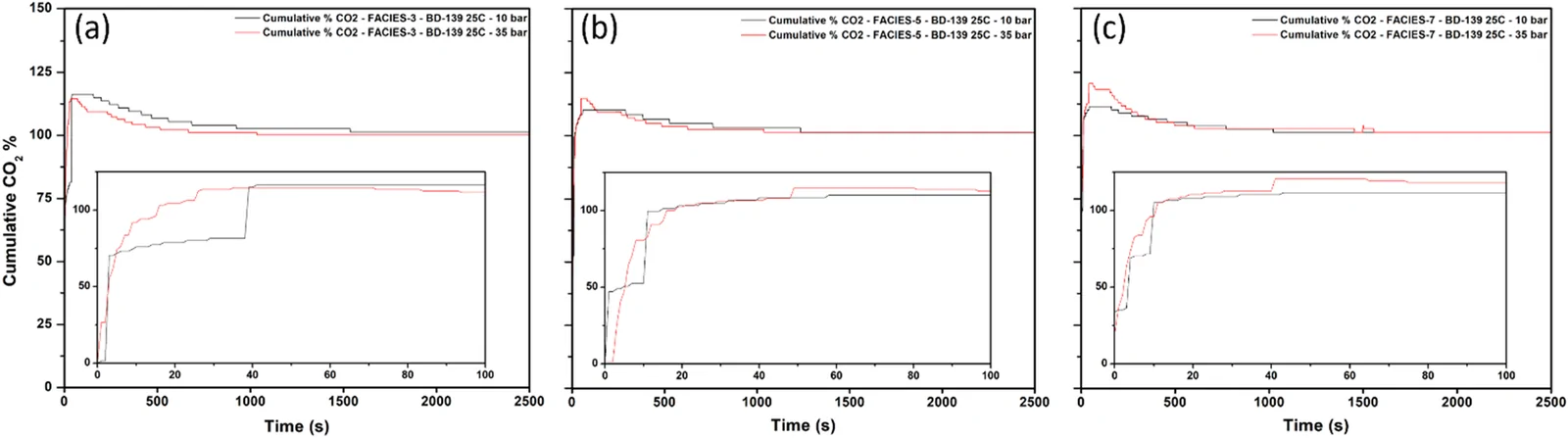
CO_2_ adsorption kinetics profiles for Facies 3, Facies 5, and Facies 7 at 25 °C under 10 and 35 bar, presented as cumulative percentage CO_2_ uptake as a function of time (min). These three facies were selected as representative high performers within Groups 1, 2, and 3, respectively. At 10 bar, equilibrium was reached in approximately 25 min (Facies 3) in figure (a), 21 min (Facies 5) in figure (b), and 16 min (Facies 7) in figure (c), with a stepped, gradual adsorption profile characteristic of diffusion-controlled uptake. At 35 bar, all three facies reached equilibrium within 15 min, exhibiting smoother and faster adsorption curves, demonstrating that elevated pressure serves as the primary kinetic driving force by increasing CO_2_ molecular flux and reducing the time to pore saturation.

## 4 Conclusions

This study systematically evaluated the CO_2_ storage-related behavior of nine sedimentary rock facies retrieved from the BD-139 well in the southeastern Michigan Basin by integrating high-pressure isochoric CO_2_ adsorption measurements with thermophysical, morphological, and mineralogical characterization. The investigated facies exhibited substantial heterogeneity in their adsorption behavior, with Group 1, and Facies 3 in particular, showing the highest apparent CO_2_ uptake, 855.7 mg/g at 35 bar and 25 °C. As set out in Section 3.5.3, this quantity exceeds the volumetric capacity of the pore system of that facies and is therefore reported as apparent isochoric uptake determined under identical conditions, establishing a relative ranking of the facies rather than an absolute storage capacity. The discrepancy between the apparent CO_2_ uptake and the relatively low N_2_–BET surface area is examined in Section 3.5.3, where it is shown to reflect the resolution limit of the measurement configuration rather than a property of the rock. The measured isosteric heats of adsorption below 60 kJ mol^–1^ are consistent with predominantly physical adsorption interactions.

Importantly, the adsorption capacities reported in this study represent intrinsic rock matrix uptake under the experimental conditions and should not be interpreted directly as field-scale storage capacities. At the reservoir scale, the total contribution of adsorptive trapping will depend on the mass and volume of each facies, bulk density, accessible pore volume, permeability, and pore connectivity, and the fraction of the measured adsorption capacity that can be accessed by CO_2_ within the reservoir. Consequently, facies with high adsorption capacities per unit mass do not necessarily provide the largest overall storage capacity if they constitute only a small fraction of the reservoir or have very limited fluid accessibility. A quantitative field-scale estimate therefore requires integration of the measured adsorption capacities with well-log/core-derived facies proportions, reservoir volume, density, porosity, and pressure–temperature conditions.

The complementary CO_2_ absorption by saline experiments demonstrates that dissolution into formation fluids represents an additional trapping mechanism that operates simultaneously with adsorption and may provide a substantially larger volumetric storage contribution in a reservoir containing significant pore-fluid volumes. In contrast, adsorption is particularly relevant to the fraction of CO_2_ that interacts directly with the accessible mineral and organic surfaces of the rock matrix. Therefore, the relative importance of dissolution and adsorption cannot be determined from the adsorption capacity alone, and it depends on the available pore-fluid volume, rock surface area, mineralogy, and reservoir architecture. The observed precipitation in the synthetic saline solution experiments further indicates the potential for subsequent mineral trapping; however, the extent and time scale of such reactions under in situ reservoir conditions require further geochemical and reactive-transport investigation.

The kinetic results provide an important constraint on the temporal behavior of the CO_2_ uptake. The initial rapid uptake followed by a gradual approach toward equilibrium indicates that a substantial fraction of the adsorption response occurs relatively quickly under the tested laboratory conditions, while subsequent redistribution and equilibration require longer times. For an actual injection scenario, however, the measured laboratory kinetics should not be interpreted as a direct reservoir-scale equilibration time because CO_2_ transport in the intact formation is additionally controlled by permeability, pore connectivity, diffusion, pressure propagation, and heterogeneity. Thus, the kinetic data are most appropriately used to define the intrinsic adsorption response and characteristic equilibration behavior of the rock matrix, while field-scale prediction requires coupling these measurements with multiphase flow and transport models.

Overall, the results demonstrate that the CO_2_ storage potential in the investigated Michigan Basin facies is governed by the combined effects of pore-volume storage, dissolution, adsorption, and potentially mineral trapping, rather than by adsorption capacity alone. The facies-resolved adsorption and kinetic data generated in this study provide experimentally constrained parameters for incorporating adsorption into reservoir-scale storage models and for evaluating the relative contributions of different trapping mechanisms. Future reservoir-scale assessment should integrate these parameters with facies distribution, porosity, permeability, formation fluid volume, and reactive-transport behavior to determine the total effective CO_2_ storage capacity and long-term retention potential of the formation.

## Supplementary Material



## Notes

Scanning electron microscopy images and energy-dispersive spectroscopy elemental maps for the nine BD-139 rock facies (Figures S1–S9); nitrogen adsorption isotherms recorded during BET surface area analysis for the nine BD-139 rock facies (Figures S10–S18) (PDF)
