# Supplementary material for: Evaluating Underground CO2 Storage Potential across Sedimentary Facies of the Michigan Basin
Source: ACS Omega. 2026 Oct 5;11(41):63827–44. doi: 10.1021/acsomega.6c08038 (PMC13689111; doi:10.1021/acsomega.6c08038)
Supplement: Supplementary file 1 [file ao6c08038_si_001.pdf]

Evaluating Underground CO_2 Storage Potential Across Sedimentary Facies of the Michigan Basin

Nimarta Kaur[a], Christina Miller[a], Pablo Sanchez[b], Emyline Kirda[c], Alberto Gutiérrez-Vega[d], Santiago Aparicio[d], Ashley Scott[e], Autumn Haagsma[e,f], Ahmad Al-Bodour[a*], Mert Atilhan[a*]

[a] *Department of Chemical and Paper Engineering, Western Michigan University, Floyd Hall, 4601 Campus Drive, Kalamazoo, MI 49008, United States*

[b] *Department of Mechanical Engineering, San Diego State University, 5500 Campanile Drive, San Diego, CA 92182, United States*

[c] *Department of Civil Engineering, University of Illinois Urbana-Champaign, 205 North Mathews Avenue, Urbana, IL 61801, United States*

[d] *Departamento de Química, Universidad de Burgos, Plaza Misael Bañuelos s/n, 09001 Burgos, Spain*

[e] *Michigan Geological Repository for Research and Education (MGRRE), Western Michigan University, 5272 West Michigan Avenue, Kalamazoo, MI 49008-5731, United States*

[f] *Michigan Geological Survey, Western Michigan University, 5272 West Michigan Avenue, Kalamazoo, MI 49008-5731, United States*

[*]Corresponding Authors: Ahmad Al-Bodour (a.al-bodour@wmich.edu) and Mert Atilhan (mert.atilhan@wmich.edu)

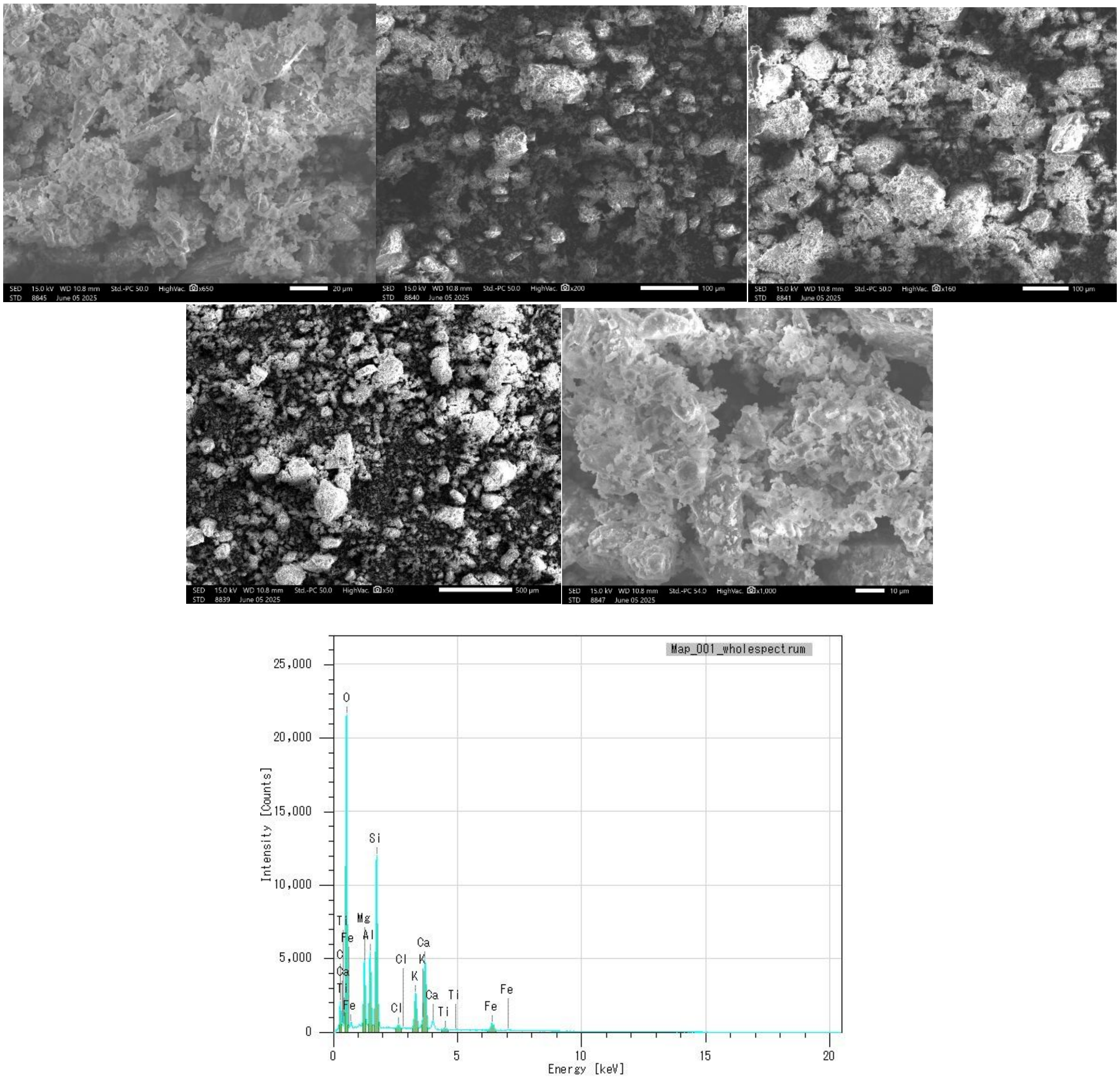

Figure S1. SEM analysis and EDS mapping for Facies 1- BD139

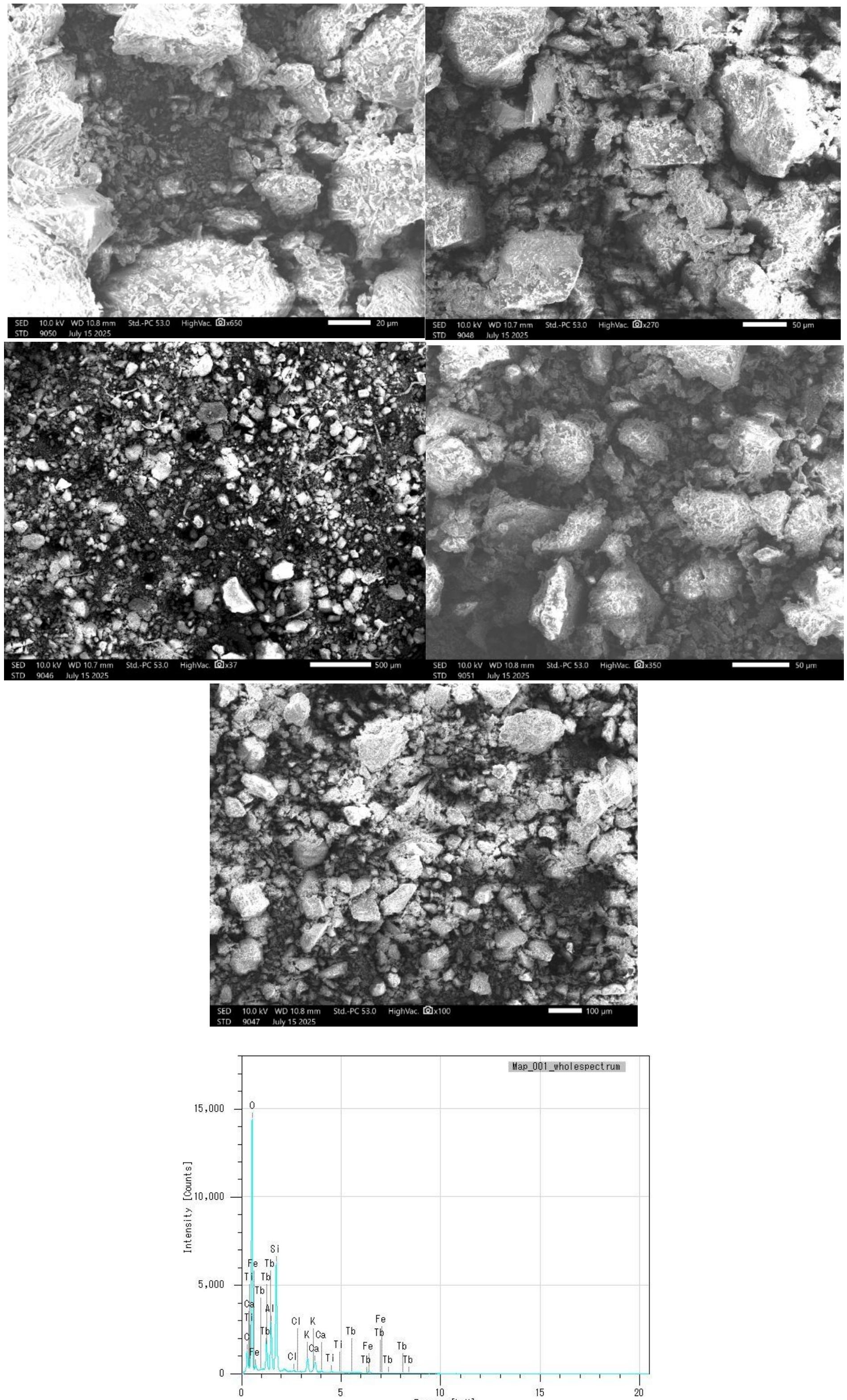

Figure S2. SEM analysis and EDS mapping for Facies 2- BD139

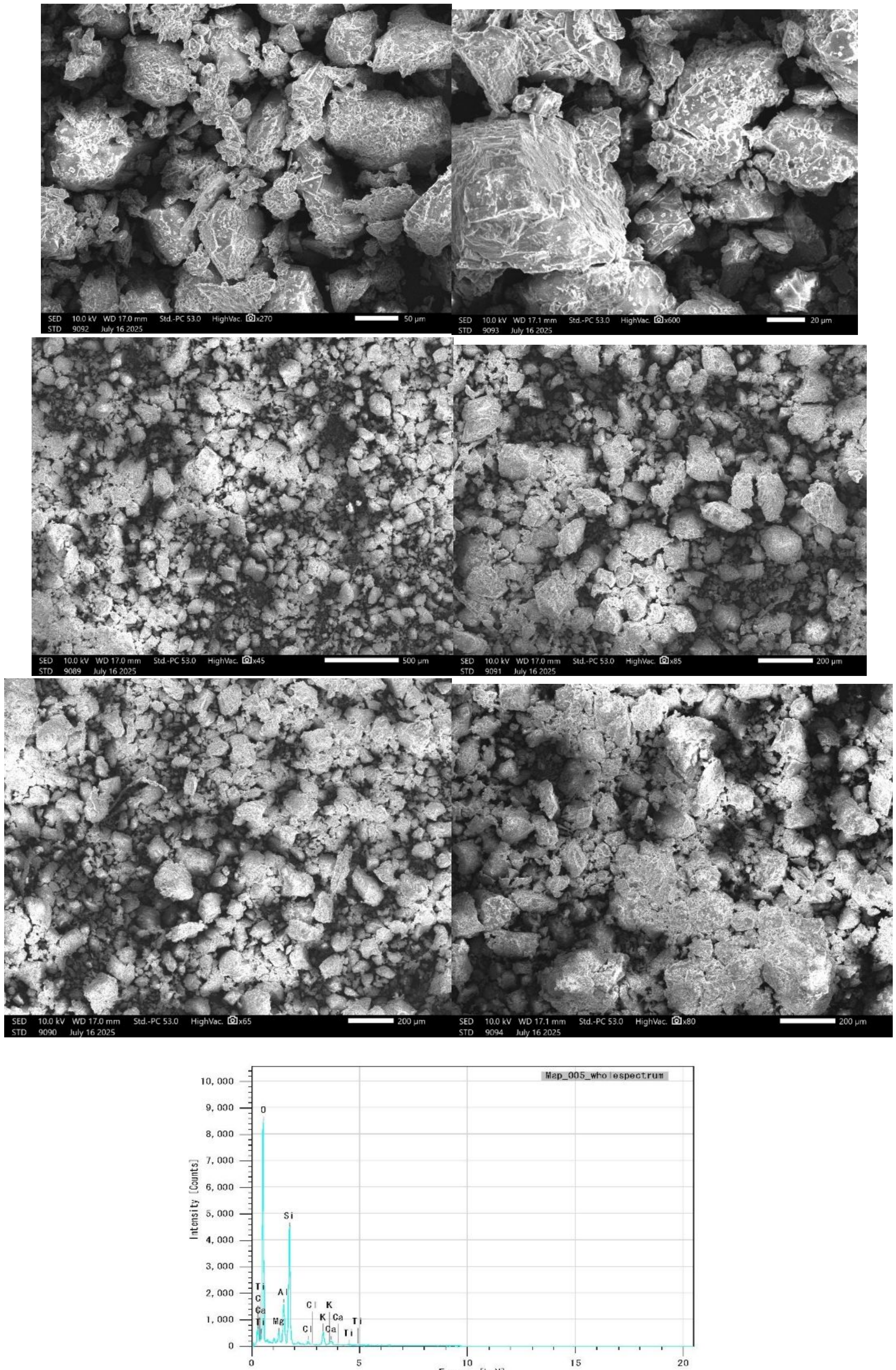

Figure S3. SEM analysis and EDS mapping for Facies 3- BD139

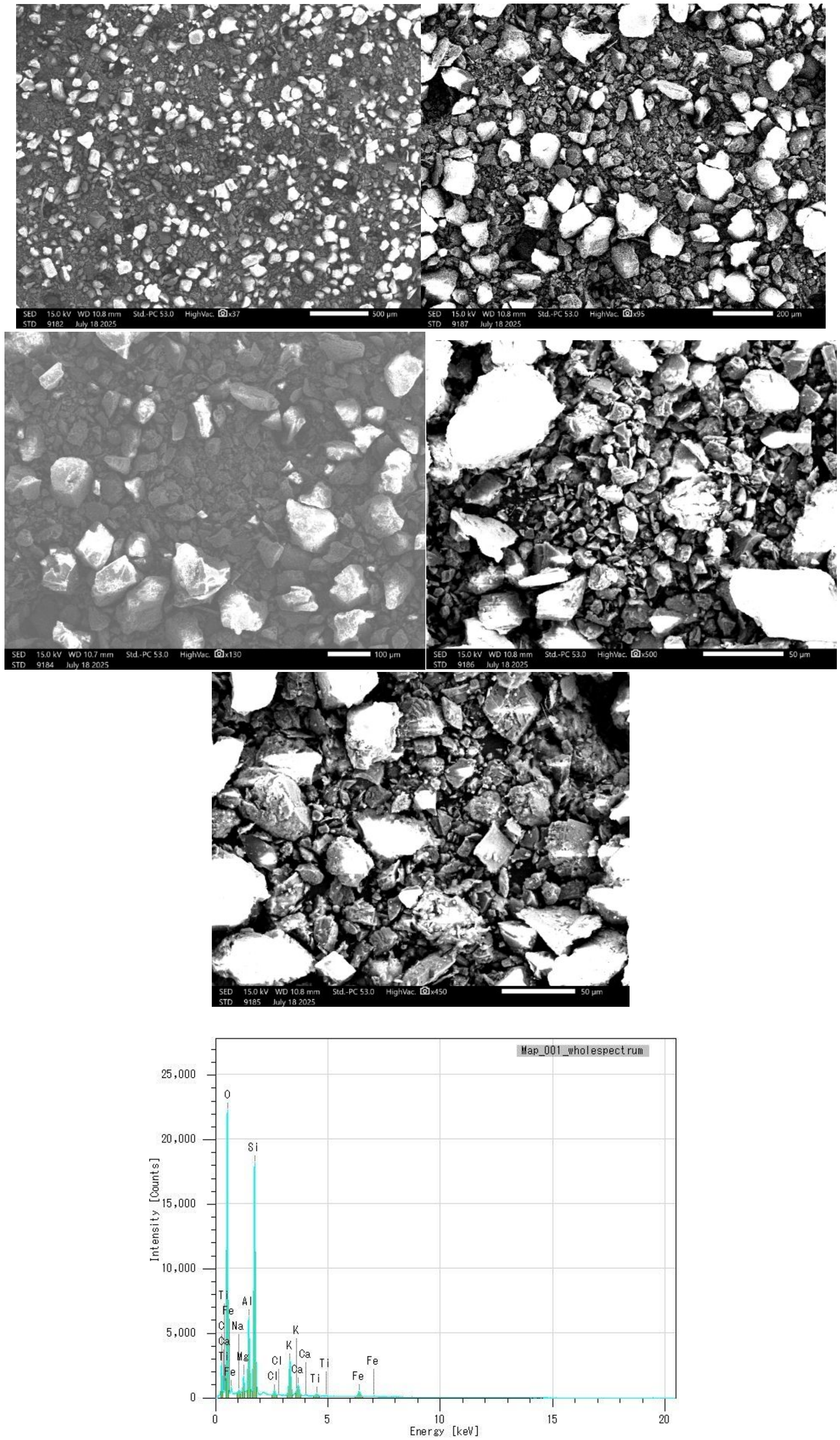

Figure S4. SEM analysis and EDS mapping for Facies 4- BD139

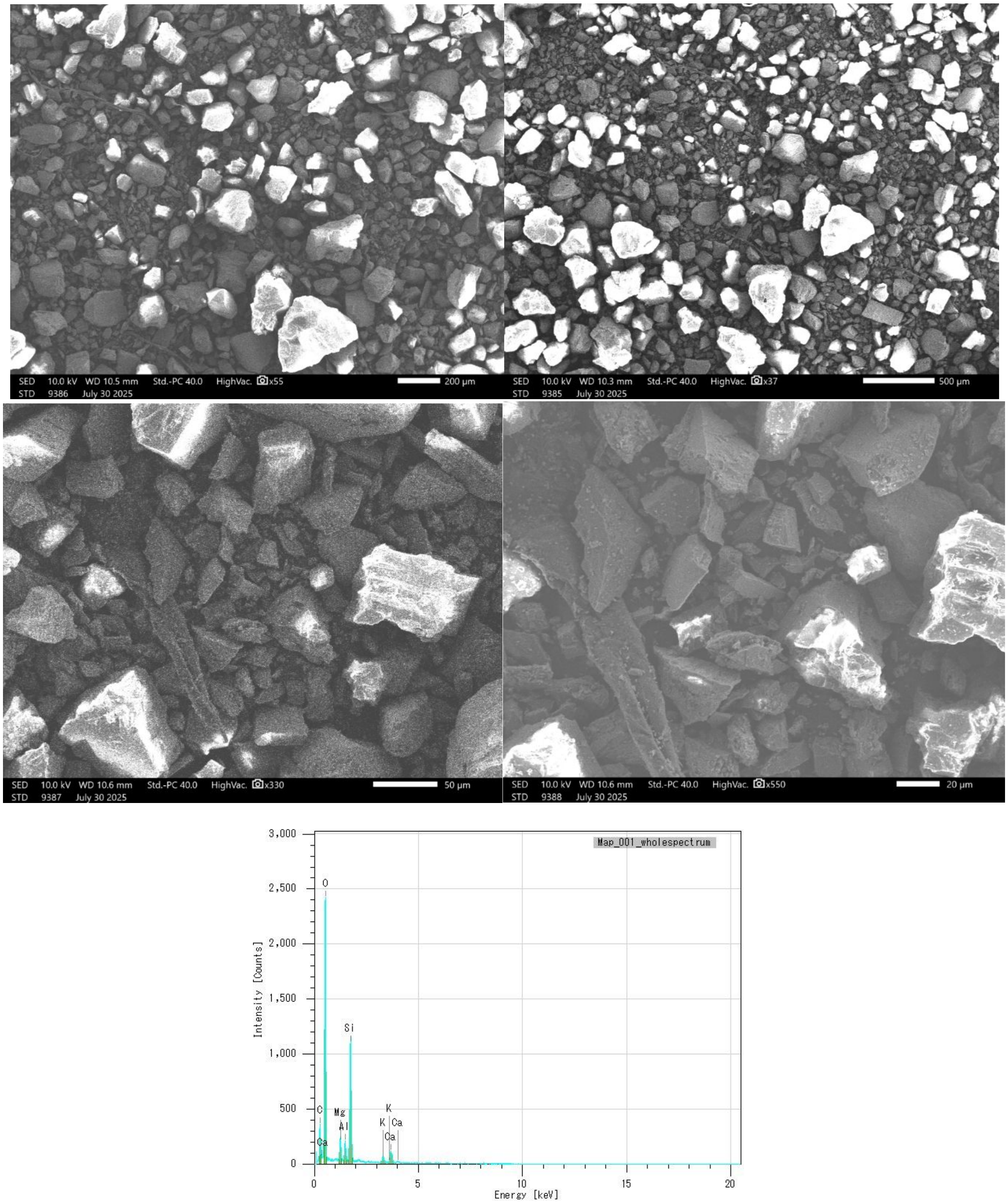

Figure S5. SEM analysis and EDS mapping for Facies 5- BD139

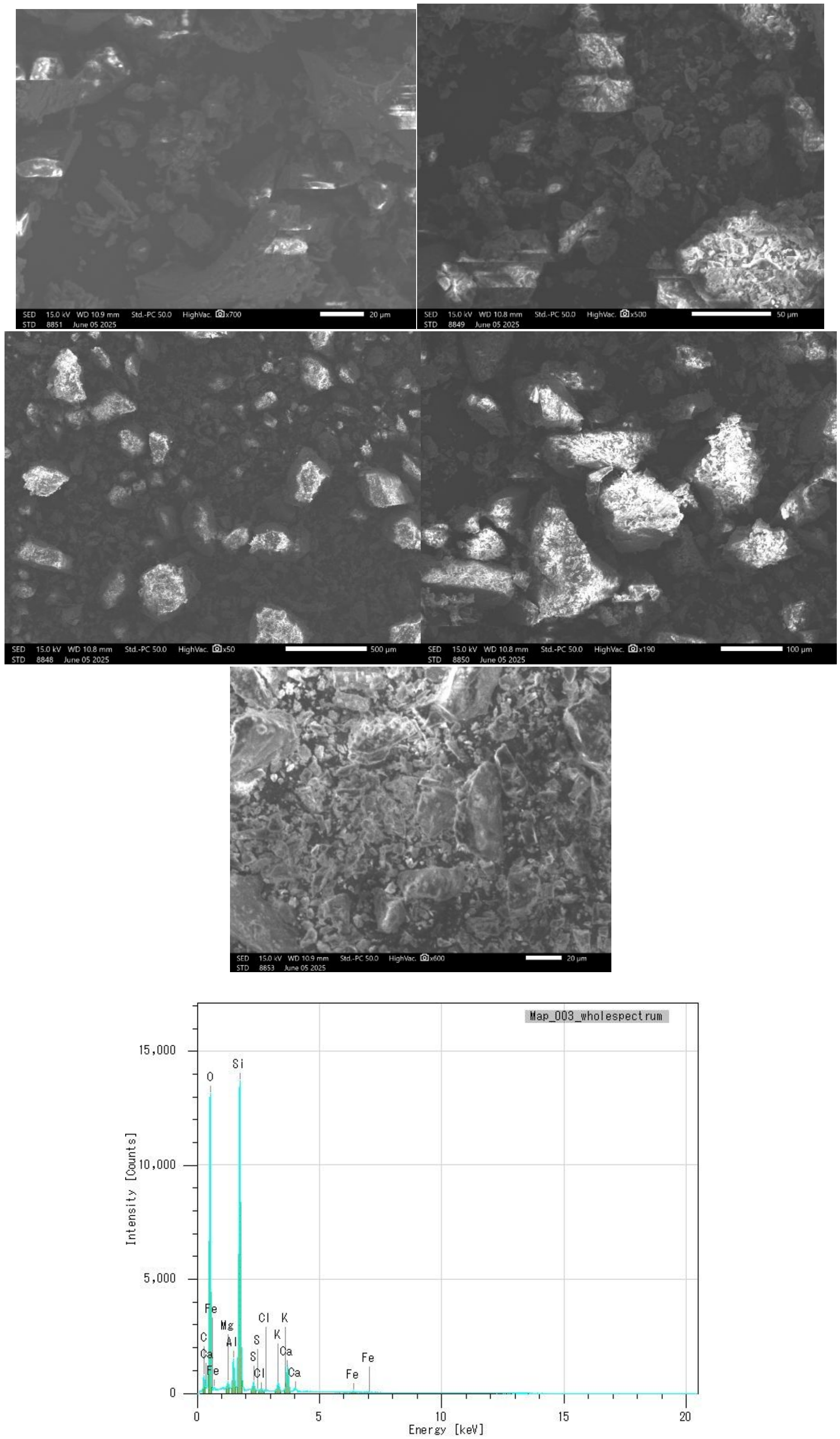

Figure S6. SEM analysis and EDS mapping for Facies 6- BD139

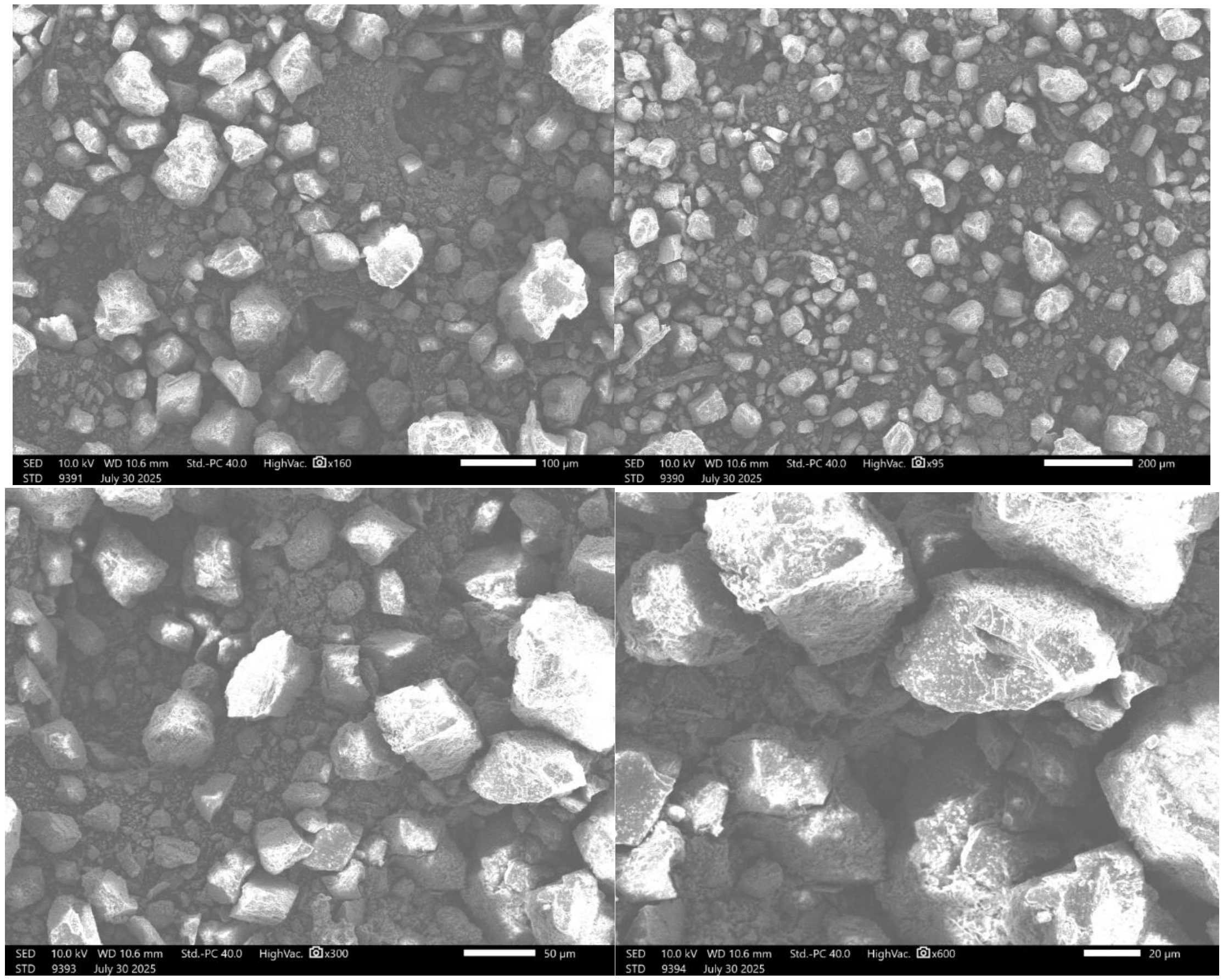

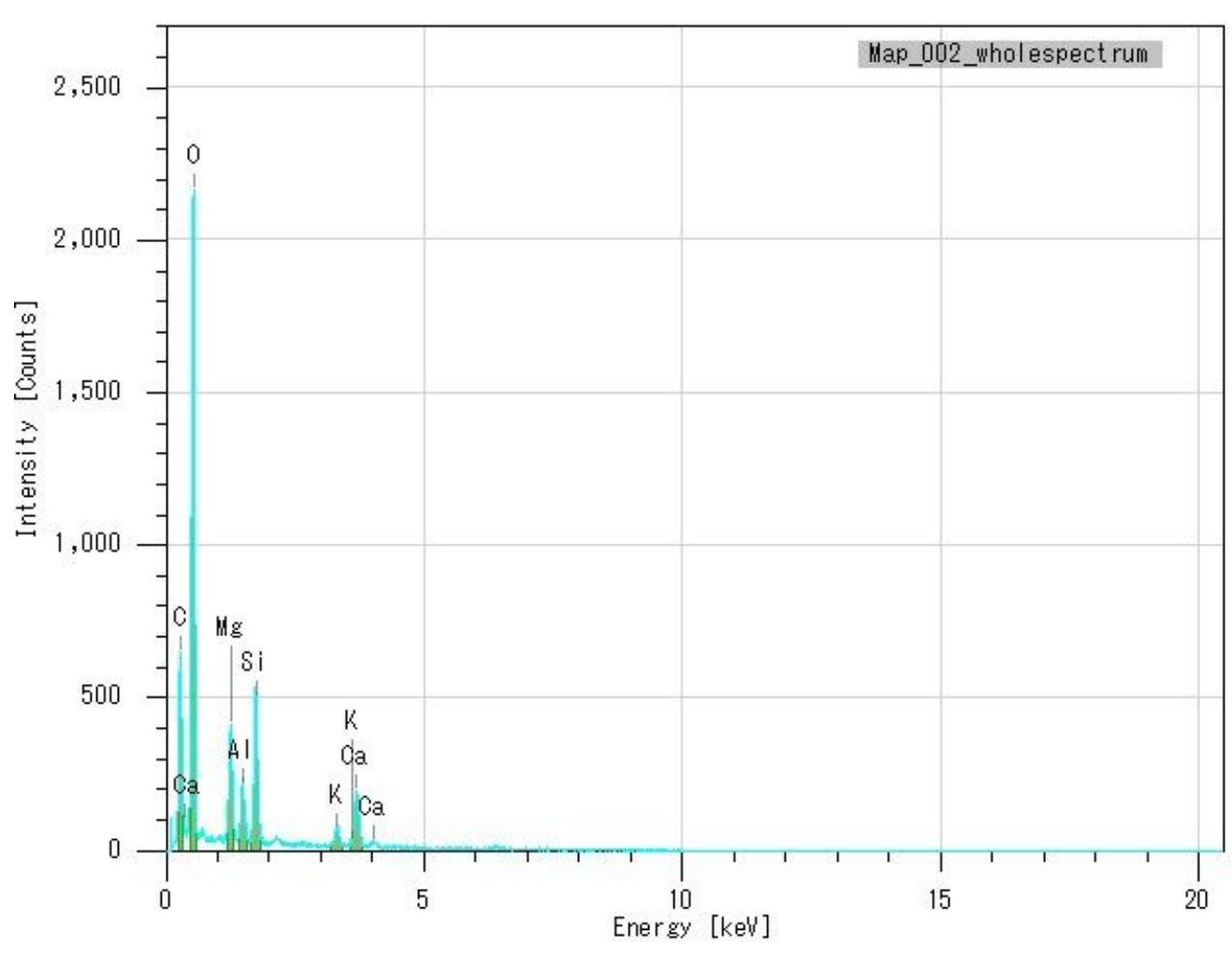

Figure S7. SEM analysis and EDS mapping for Facies 7- BD139

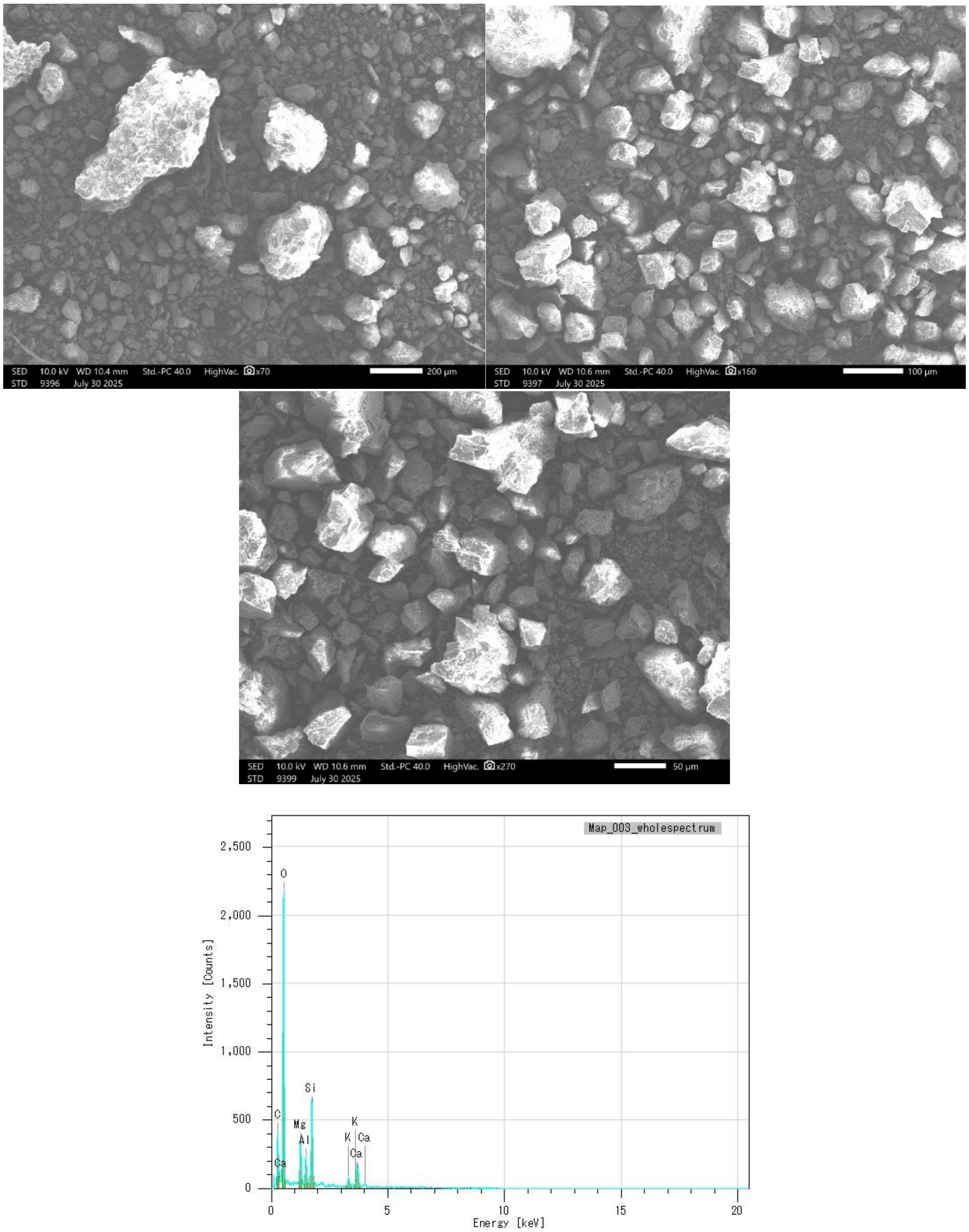

Figure S8. SEM analysis and EDS mapping for Facies 8- BD139

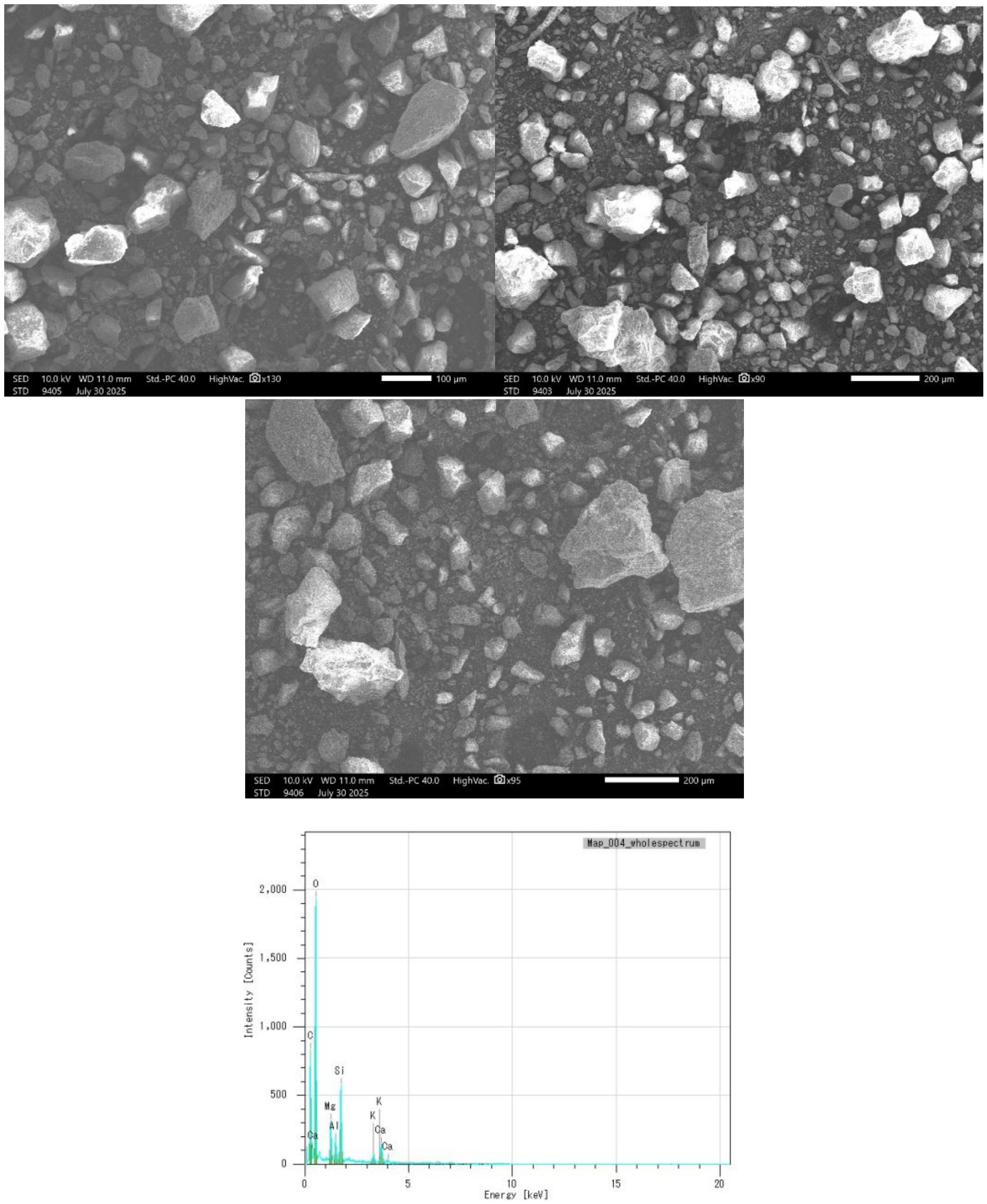

Figure S9. SEM analysis and EDS mapping for Facies 9- BD139

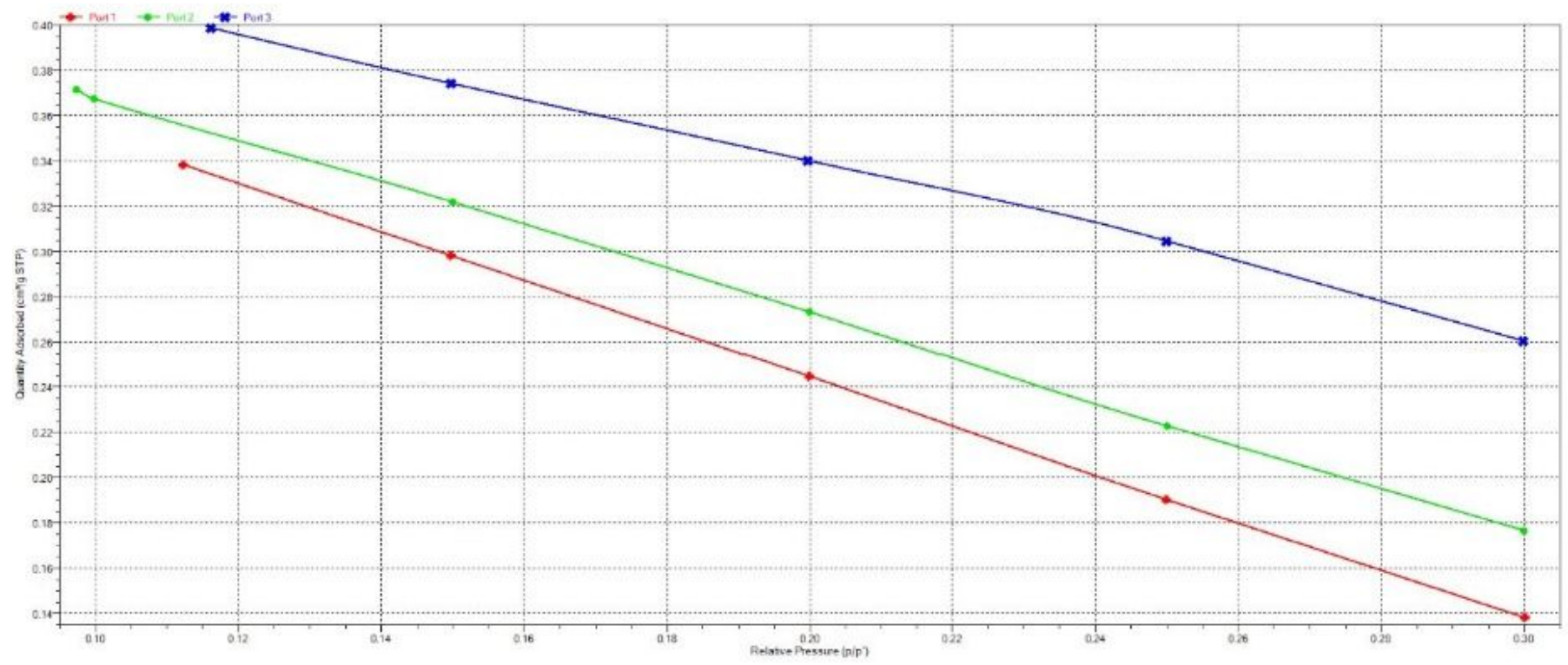

Figure S10 - N_2 Adsorption by Facies 1 during the (BET) surface area analysis

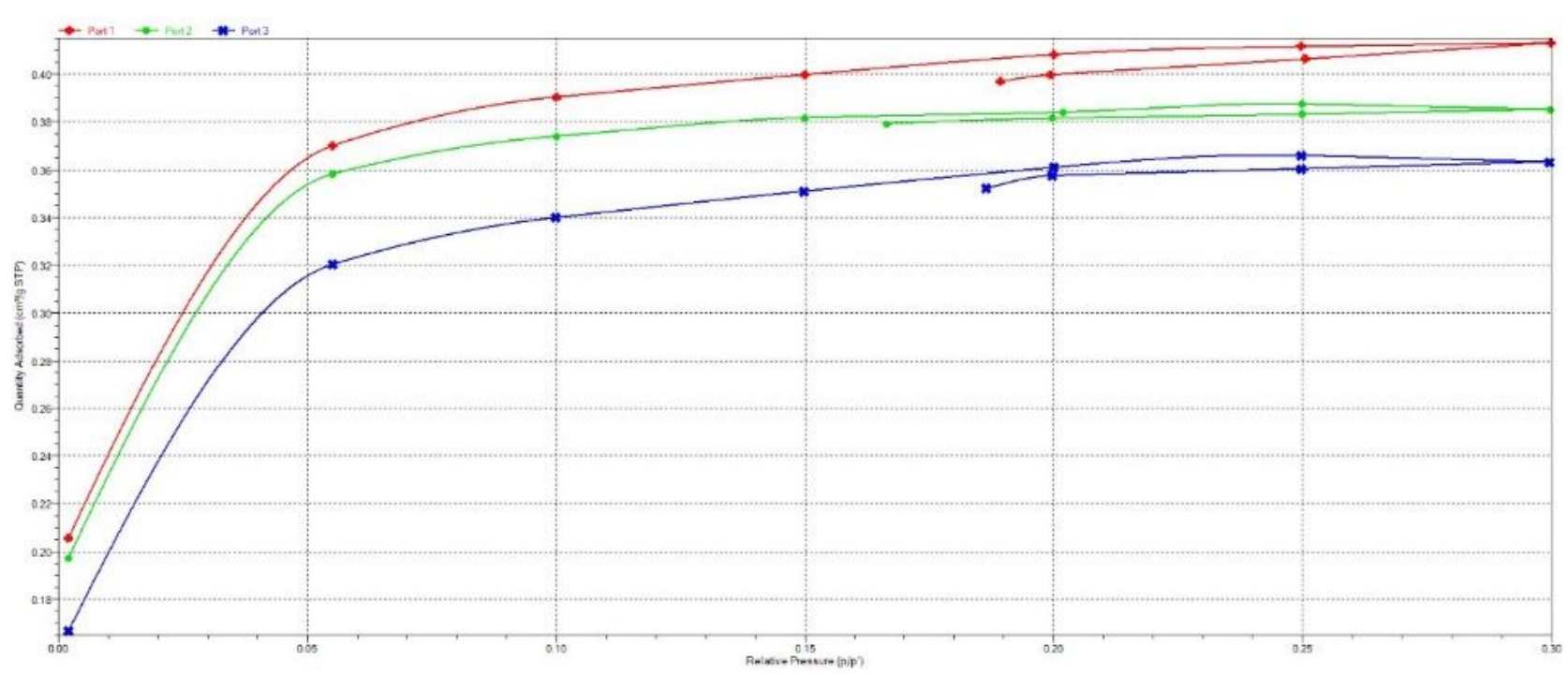

Figure S11 - N_2 Adsorption by Facies 2 during the (BET) surface area analysis

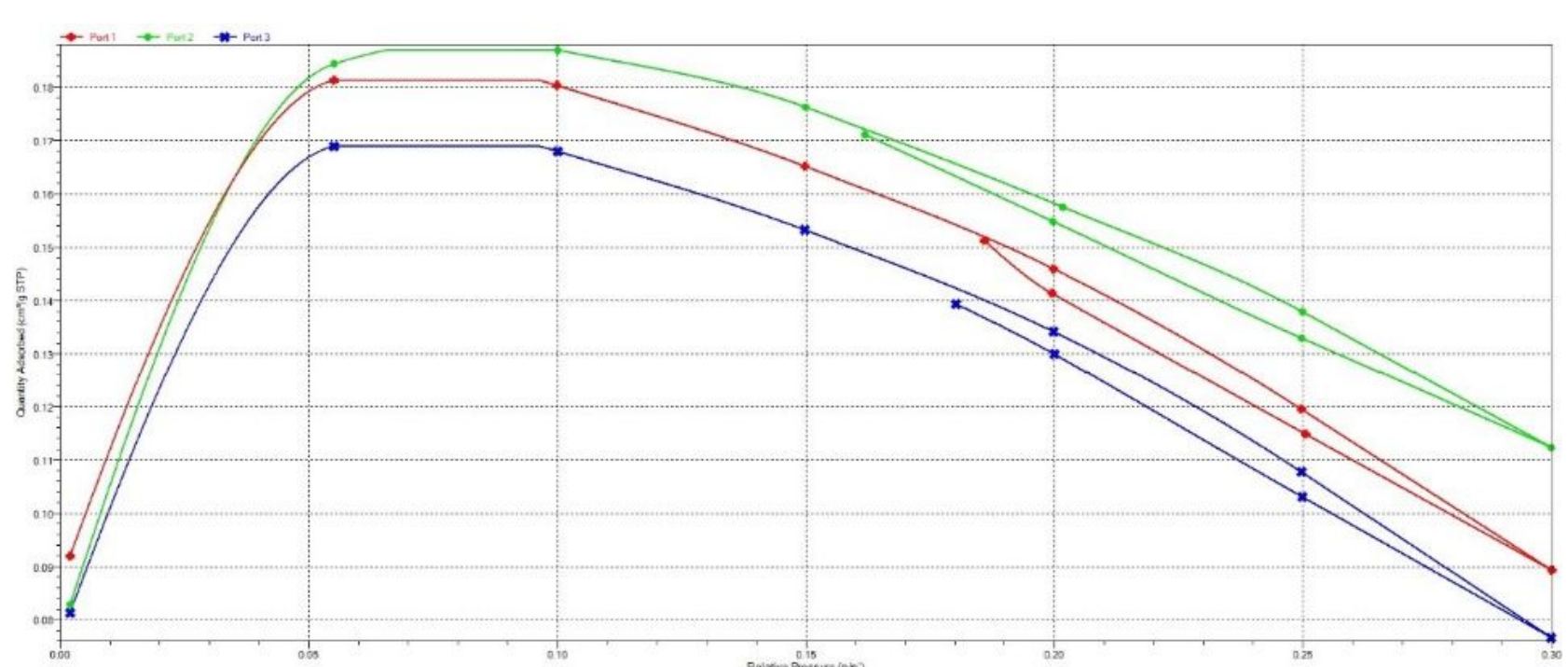

Figure S12 - N_2 Adsorption by Facies 3 during the (BET) surface area analysis

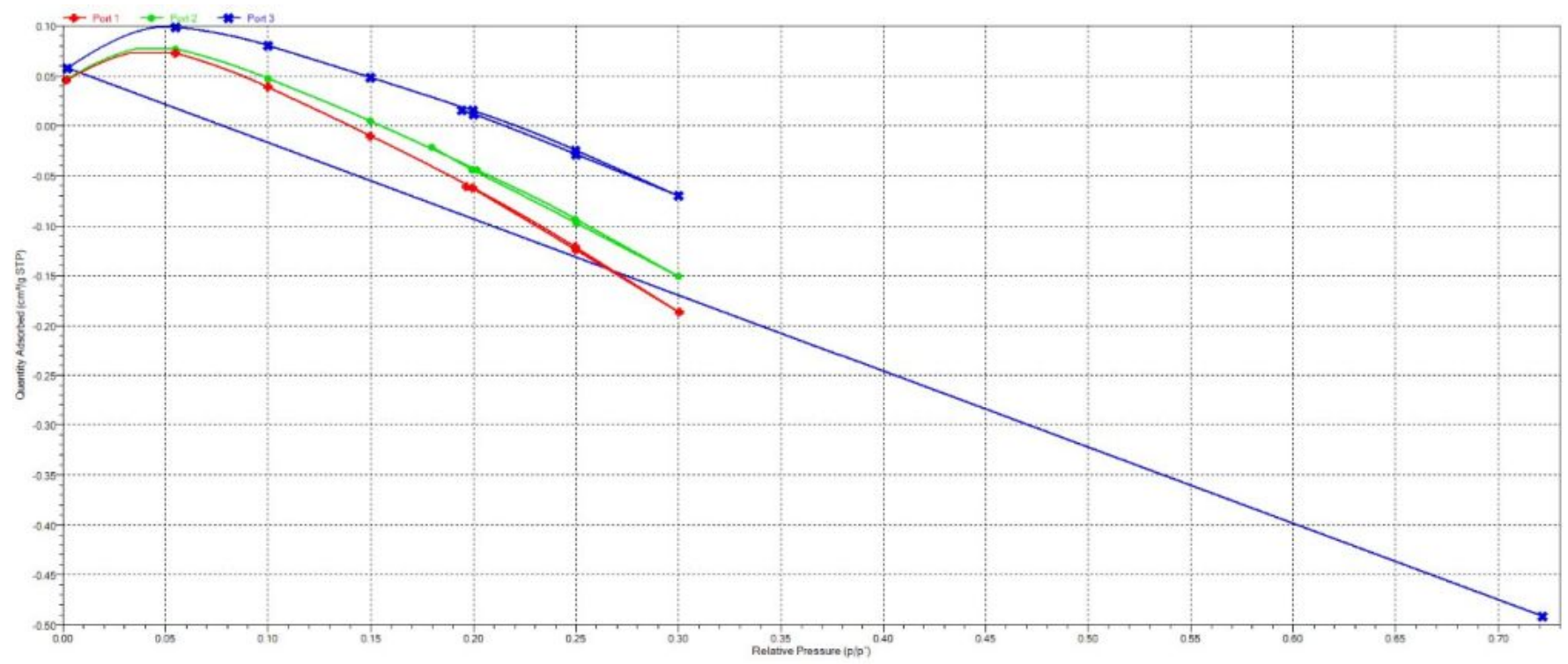

Figure S13 - N_2 Adsorption by Facies 4 during the (BET) surface area analysis

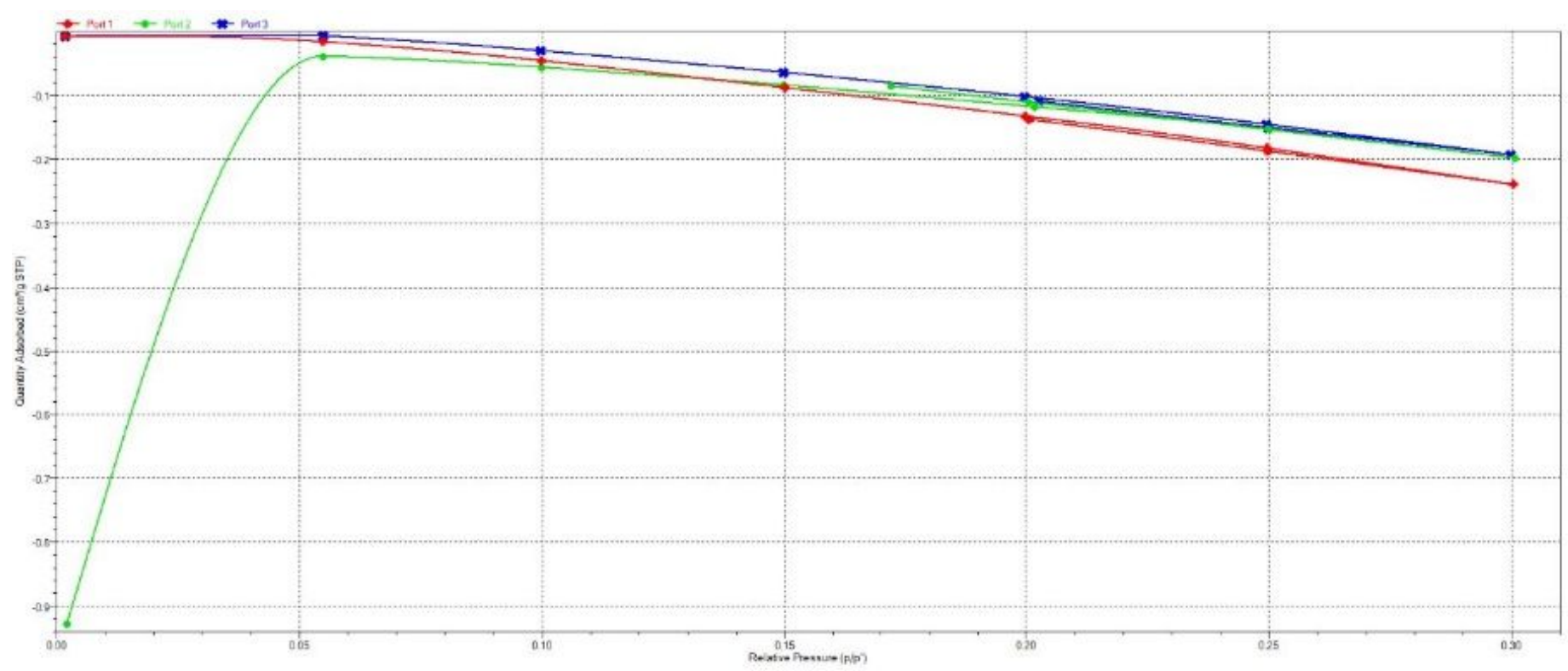

Figure S14 - N_2 Adsorption by Facies 5 during the (BET) surface area analysis

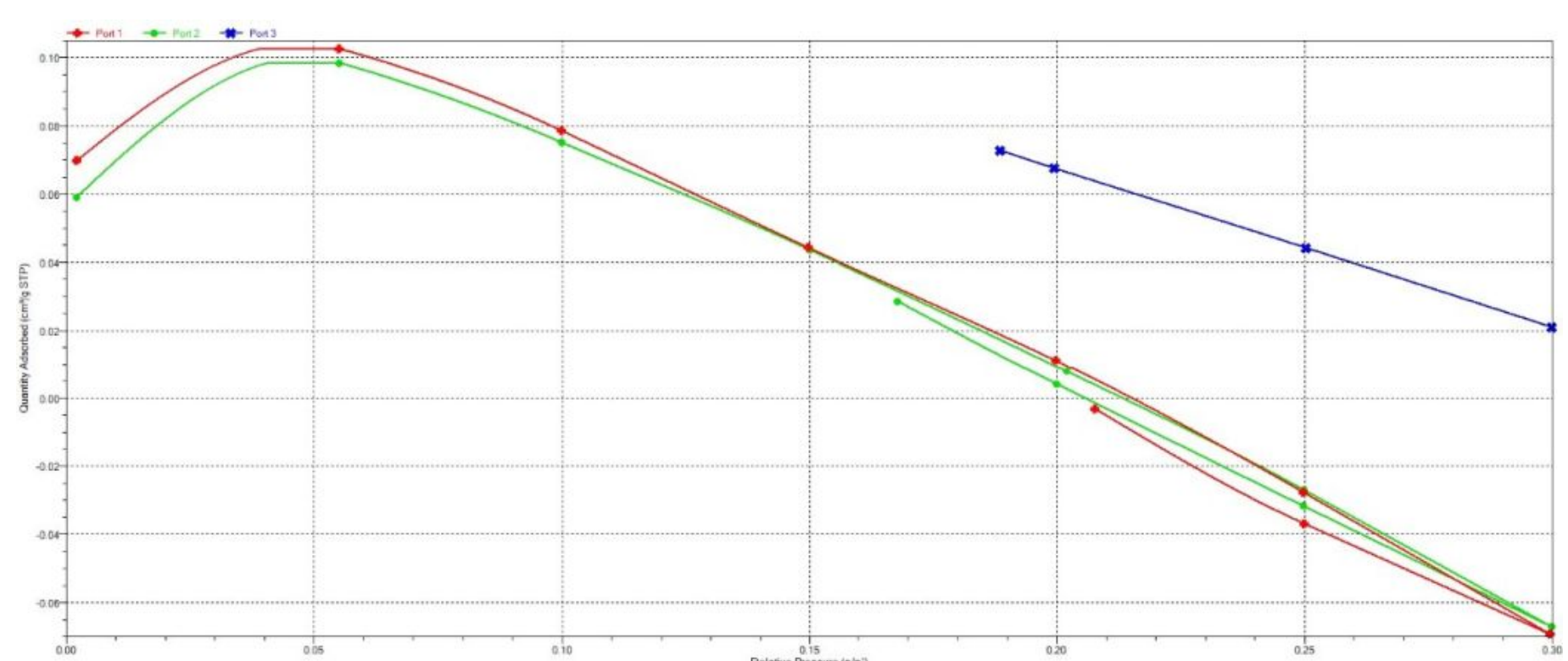

Figure S15 - N_2 Adsorption by Facies 6 during the (BET) surface area analysis

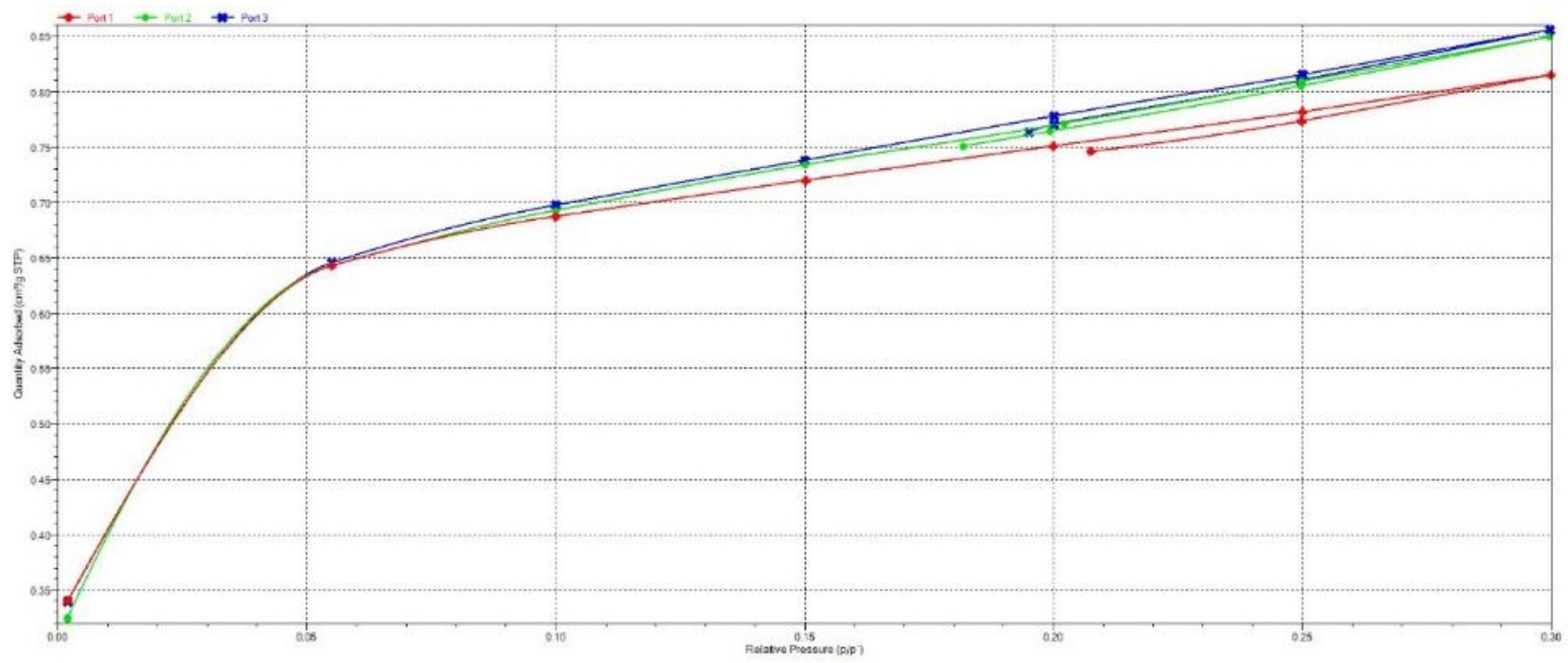

Figure S16 - N_2 Adsorption by Facies 7 during the (BET) surface area analysis

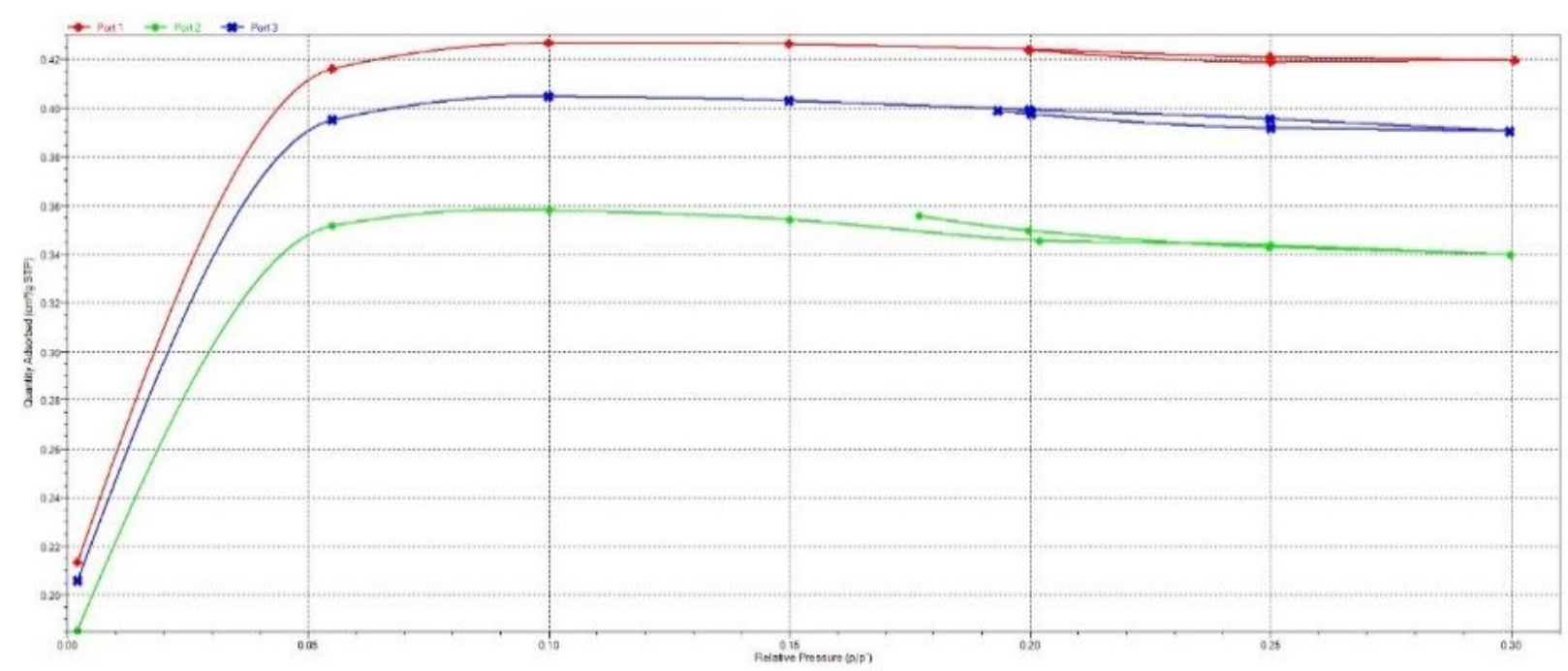

Figure S17 - N_2 Adsorption by Facies 8 during the (BET) surface area analysis

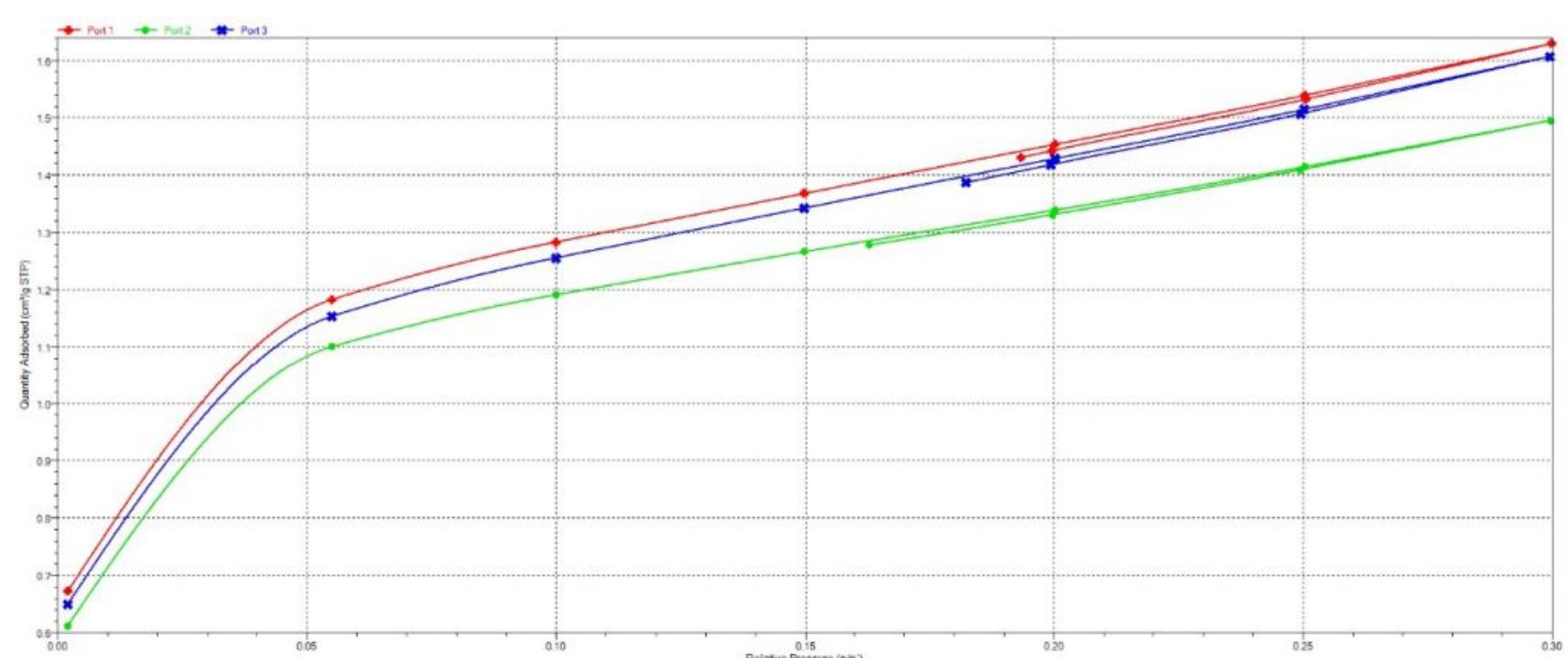

Figure S18 - N_2 Adsorption by Facies 9 during the (BET) surface area analysis
